# Blood vessel control of macrophage maturation promotes arteriogenesis in ischemia

**DOI:** 10.1038/s41467-017-00953-2

**Published:** 2017-10-16

**Authors:** Kashyap Krishnasamy, Anne Limbourg, Tamar Kapanadze, Jaba Gamrekelashvili, Christian Beger, Christine Häger, Vladimir J. Lozanovski, Christine S. Falk, L. Christian Napp, Johann Bauersachs, Matthias Mack, Hermann Haller, Christian Weber, Ralf H. Adams, Florian P. Limbourg

**Affiliations:** 1 Vascular Medicine Research, Hannover Medical School, Hannover, D 30625 Germany; 2 Department of Nephrology and Hypertension, Hannover Medical School, Hannover, D 30625 Germany; 3 Integrated Research and Treatment Center Transplantation, Hannover Medical School, Hannover, D 30625 Germany; 4 Institute of Transplant Immunology, Hannover Medical School, Hannover, D 30625 Germany; 5 Department of Cardiology, Hannover Medical School, Hannover, D 30625 Germany; 6 Department of Nephrology, Uniklinikum, Regensburg, D 93053 Germany; 7 Institute for Cardiovascular Prevention, and German Centre for Cardiovascular Research (DZHK), partner site Munich Heart Alliance, Ludwig-Maximilians-University, Munich, D 80336 Germany; 8 Cardiovascular Research Institute Maastricht (CARIM), Maastricht, 6229 ER The Netherlands; 9 Department of Tissue Morphogenesis, Faculty of Medicine, Max-Planck-Institute for Molecular Biomedicine, and University of Münster, Münster, D 48149 Germany; 10 Present Address: Department of Plastic, Aesthetic, Hand and Reconstructive Surgery, Hannover Medical School, Hannover, D 30625 Germany; 11 Present Address: Institute for Laboratory Animal Science and Central Animal Facility, Hannover Medical School, Hannover, D 30625 Germany; 12 Present Address: Department of General and Transplant Surgery, University Hospital Heidelberg, Heidelberg, D 69117 Germany

## Abstract

Ischemia causes an inflammatory response that is intended to restore perfusion and homeostasis yet often aggravates damage. Here we show, using conditional genetic deletion strategies together with adoptive cell transfer experiments in a mouse model of hind limb ischemia, that blood vessels control macrophage differentiation and maturation from recruited monocytes via Notch signaling, which in turn promotes arteriogenesis and tissue repair. Macrophage maturation is controlled by Notch ligand *Dll1* expressed in vascular endothelial cells of arteries and requires macrophage canonical Notch signaling via *Rbpj*, which simultaneously suppresses an inflammatory macrophage fate. Conversely, conditional mutant mice lacking *Dll1* or *Rbpj* show proliferation and transient accumulation of inflammatory macrophages, which antagonizes arteriogenesis and tissue repair. Furthermore, the effects of Notch are sufficient to generate mature macrophages from monocytes ex vivo that display a stable anti-inflammatory phenotype when challenged with pro-inflammatory stimuli. Thus, angiocrine Notch signaling fosters macrophage maturation during ischemia.

## Abstract (web summary)

Molecular mechanisms of macrophage-mediated regulation of artery growth in response to ischemia are poorly understood. Here the authors show that vascular endothelium controls macrophage maturation and differentiation via Notch signaling, which in turn promotes arteriogenesis and ischemic tissue recovery.

## Introduction

Growth of functional arteries is essential for the restoration of blood flow to ischemic limbs and organs. Ischemia causes an inflammatory response that is intended to initiate restoration of perfusion and tissue repair yet often aggravates damage or disturbs healing^1, 2^. Monocytes and macrophages in particular are recruited to ischemic vessels and tissues and are essential for remodeling of small collateral vessels into conduit arteries, or arteriogenesis, a process capable of fully restoring blood flow^3^.

Macrophages have diverse phenotypes and functions in different contexts, ranging from pro-inflammatory states with detrimental effects on tissues in host defense to trophic or reparative roles, as described in arteriogenesis^4, 5^. Macrophage diversity is considered to arise from combinations of differentiation and activation or polarization programs^6^. Differentiation programs induce a stable and irreversible transition and maturation from macrophage precursors to distinct macrophage lineages under the control of external lineage factors, such as macrophage colony stimulating factor (CSF1) or granulocyte-macrophage CSF (CSF2)^7^. These cooperate with mostly unknown tissue-specific identity signals^6^, which can ultimately lead to terminal macrophage differentiation mediated by Ets repressor Etv3 (METS), which induces cell cycle arrest by suppressing Myc^8^. In contrast, activation programs induce mostly reversible changes in response to cytokines or danger signals^6^. This plasticity was initially termed polarization^9^, describing essentially pro- and anti-inflammatory fates, but was recently revised into a spectrum model of activation, recognizing that the number of stimuli and resultant activation programs is not dichotomous, but rather more diverse^10^. In a model of myocardial infarction, it was recently shown that the subset of Ly6C^hi^ monocytes is recruited to ischemic tissues and differentiates into macrophages, which is required for cardiac healing, since impaired differentiation lead to adverse cardiac remodeling^11^. Exactly how macrophage differentiation and function is regulated during ischemia and arteriogenesis still remains largely unknown.

Organ-specific endothelial cells (EC) exert angiocrine functions that are involved in homeostasis and tissue regeneration independent of blood flow^12^. After injury, instructive cues expressed by local EC orchestrate the injury response of tissue-resident or hematopoietic progenitor cells and fibroblasts during organ regeneration, which in part is mediated by endothelial-specific expression of Notch ligands such as Jagged-1^13–15^.

Notch signaling is a cell to cell contact-dependent signaling pathway regulating vascular development, branching morphogenesis, and homeostasis^16–18^. Activation of Notch receptors is controlled by membrane-bound Notch ligands of the Jagged (*Jag*) and Delta-like (*Dll*) gene families, which show different Notch receptor binding affinities and tissue expression patterns, thereby controlling specific Notch signaling outcomes^19^. The Notch ligand *Dll1* is selectively expressed in vascular EC, and *Dll1* haploinsufficiency leads to severely impaired arteriogenesis and ischemic tissue damage in a mouse model of hind limb ischemia^20^. We recently described that Notch signaling activated by endothelial *Dll1* regulates cell fate of monocyte subsets under steady state conditions^21^.

We now tested the hypothesis that endothelial *Dll1* mediates angiocrine effects in arteriogenesis by influencing macrophages in a mouse model of hind limb ischemia^22^. We show that endothelial *Dll1* regulates macrophage differentiation and maturation from invading Ly6C^hi^ monocytes, which promotes arteriogenesis and tissue repair after ischemia.

## Results

### Macrophages develop from Ly6C^hi^ monocytes in ischemia

**Fig. 1:**
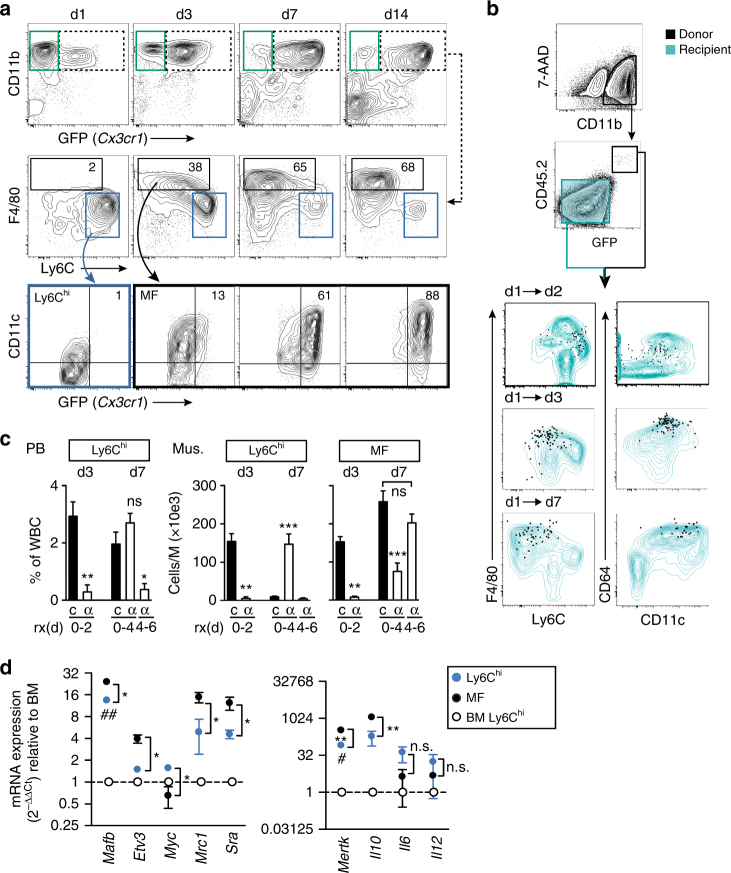
Origin of macrophages in ischemia. **a** Representative flow cytometric analysis of ischemic tibialis anterior muscle of *Cx3cr1* ^*GFP/+*^ mice. Granulocytes (green), Ly6C^hi^ monocytes (blue) and macrophages (black), *n* = 3 mice/group. **b** Adoptive cell transfer at d1 after HLI and cell fate tracking of CD45.2^+^CD11b^+^GFP^+^Ly6C^hi^ monocytes transferred into CD45.1^+^ recipients. Representative flow cytometry of recipient muscle, replicated at least three times. **c** Treatment with CCR2 blocking antibody MC-21 (α) or isotype control **c** for indicated time intervals (r × d) after HLI and analysis of cell populations in peripheral blood (PB) and muscle (M) by flow cytometry. *n* = 5/7/7 mice/group, error bars represent s.e.m. ***p < 0.001, **p < 0.01, *p < 0.05 *indicates comparison between treatment with MC-21 (α) vs isotype control **c** by one way ANOVA with Bonferroni multiple comparison test. **d** Quantitative RT-PCR analysis of cell populations of *Cx3cr1* ^*GFP/+*^ mice sorted from d3 ischemic muscle relative to bone marrow (BM) Ly6C^hi^ monocytes. *n* = 3 independent experiments, error bars represent s.e.m. ***p < 0.001, **p < 0.01, *p < 0.05, ^#^Comparison between muscle and BM Ly6C^hi^ cells, *Comparison between muscle Ly6C^hi^ and muscle macrophages (MF), one way ANOVA with Bonferroni multiple comparison post-test

To study the origin and regulation of macrophages in response to peripheral tissue ischemia, we first characterized the population dynamics of monocytes and macrophages by flow cytometry (for gating and antibody panel see Supplementary Fig. 1 and Supplementary Table 1) in the mouse model of hind limb ischemia (HLI)^22^. We analyzed *Cx3cr1* ^*GFP/+*^ reporter mice^23^, in which monocyte and macrophages, but not granulocytes, express distinct intensities of GFP (mouse strain information see Supplementary Table 2). Ischemia triggered recruitment of Ly6C^hi^ monocytes into muscle, which was closely followed by the appearance of macrophages (Fig. 1a, Supplementary Fig. 1). While the monocyte response was transient, macrophage development was more sustained and characterized by gradual down regulation of Ly6C and up regulation of Cx3cr1, CD11c, and MHC class II over time, suggesting maturation of macrophages from monocytes.

After myocardial infarction, cardiac macrophages are reported to develop from Ly6C^hi^ monocytes and mediate healing^11^. To confirm and extend these findings we performed adoptive transfer studies with Ly6C^hi^ monocytes that were isolated from CD45.2^+^ *Cx3cr1* ^*GFP/+*^ mice (Supplementary Fig. 2a) and intravenously transferred into CD45.1^+^ recipient mice 1 day after induction of hindlimb ischemia. Cell fate of donor cells, distinguished from recipient by expression of GFP and congenic CD45, was analyzed by flow cytometry 1, 2, and 6 days after transfer in ischemic limb muscle. Transferred Ly6C^hi^ monocytes gradually and uniformly adopted a macrophage phenotype similar to endogenous macrophages, displaying gradual downregulation of Ly6C and upregulation of F4/80, Fcγ receptor CD64 and CD11c integrin over time (Fig. 1b, Supplementary Fig. 2b). Thus, in ischemic muscle Ly6C^hi^ monocytes give rise to macrophages, which continue to undergo phenotypic changes suggesting macrophage maturation over time. In contrast, donor monocytes retrieved from the spleen of recipient mice predominantly adopted a Ly6C^lo^ monocyte phenotype, as previously reported^21^, which was not even transiently observed in muscle (Supplementary Fig. 2c, d).

Our data so far suggested that, in ischemic muscle, a wave of early invading Ly6C^hi^ monocytes gives rise to macrophages that gradually change their phenotype as they differentiate. We therefore tested the effects of Ly6C^hi^ monocyte-depletion on the development of ischemic macrophages using anti-CCR2 antibody treatment (clone MC-21), which selectively and transiently depletes Ly6C^hi^ monocytes^24^. We specifically addressed the effects of early vs. delayed monocyte depletion on the kinetics of macrophage development after ischemia. With an early depletion strategy (d0–d4 after HLI), MC-21 treatment strongly depleted Ly6C^hi^ monocytes from peripheral blood (PB) and muscle at day 3 and also dramatically reduced the number of macrophages at this time point (Fig. 1c). At d7, Ly6C^hi^ monocyte levels in PB recovered, reflecting the transient nature of monocyte depletion, while Ly6C^hi^ monocyte numbers in muscle were increased but numbers of macrophages were still strongly reduced compared with isotype control (Fig. 1c). In contrast, delayed antibody treatment (from d4 onwards) reduced the number of Ly6C^hi^ monocytes in PB, but did not significantly affect the number of ischemic macrophages at day 7. Thus, while initial depletion of Ly6C^hi^ monocytes impairs early and late macrophage populations, delayed monocyte depletion does not influence macrophage development. These data clearly demonstrate that early invading Ly6C^hi^ monocytes give rise to ischemic macrophages, which continue to change their phenotype as they develop over time.

We next characterized the gene expression changes occurring during monocyte-to-macrophage differentiation in ischemic muscle. We isolated Ly6C^hi^ monocytes and macrophages from ischemic muscle and compared the gene expression signatures with naive Ly6C^hi^ monocytes from the bone marrow (BM) of the same mouse. Compared with BM Ly6C^hi^ monocytes, ischemic muscle Ly6C^hi^ monocytes showed significant upregulation of transcription factor *Mafb*, involved in macrophage differentiation from precursors, and the macrophage marker Mer tyrosine kinase receptor (*Mertk*) (Fig. 1d)^25^. On the other hand, compared with muscle monocytes, ischemic macrophages showed significantly elevated levels of genes involved in macrophage differentiation (*Mafb*, Scavenger receptor *Sra*) and maturation, indicated by suppression of *Myc* but high expression of the transcription factor *Etv3*, which regulates terminal macrophage differentiation by suppressing *Myc* (Fig. 1d)^8^. At the same time, macrophages showed upregulation of Mannose receptor *Mrc1* and *Mertk*, and high expression of anti-inflammatory *Il10* and low expression of inflammatory cytokines *Il6* and *Il12*, a profile consistent with reparative macrophages. Altogether, these data demonstrate that ischemic macrophages develop from Ly6C^hi^ monocytes by gradual differentiation and maturation.

### Dll1 promotes macrophage differentiation

**Fig. 2:**
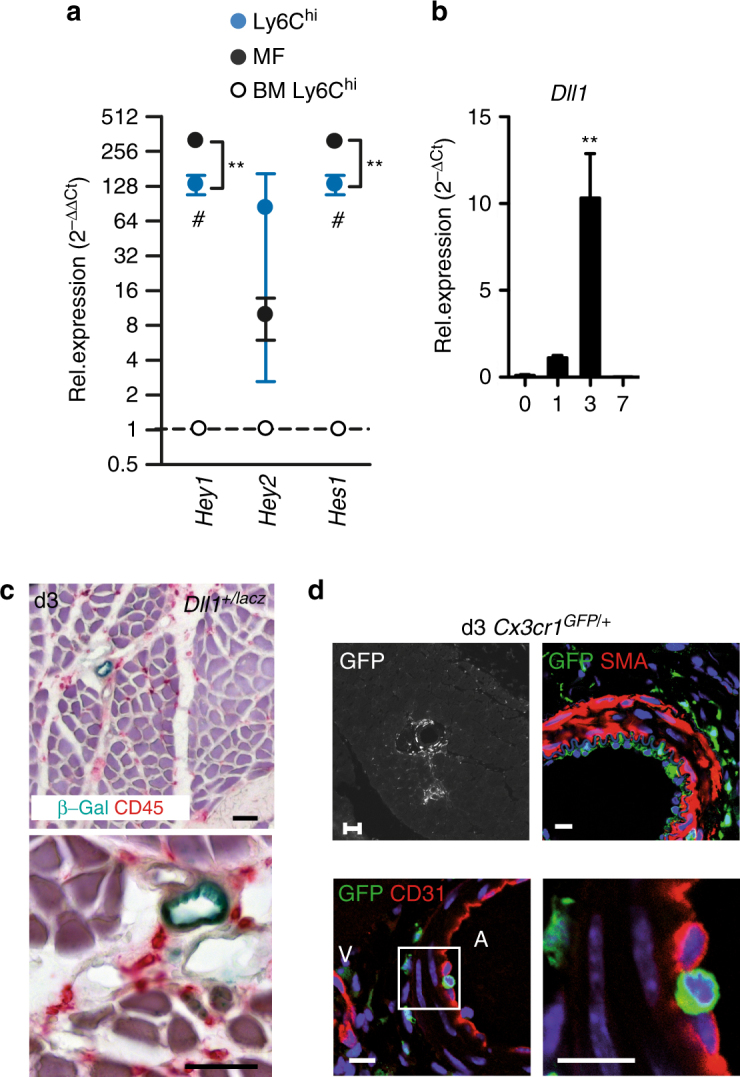
Ischemia triggers induction of Notch ligand Dll1 in arteries. **a** Quantitative RT-PCR analysis of cell populations of *Cx3cr1* ^*GFP/+*^ mice sorted from d3 ischemic muscle relative to bone marrow (BM) Ly6C^hi^ monocytes. *n* = 3 independent experiments, error bars represent s.e.m. ***p < 0.001, **p < 0.01, *p < 0.05 ^#^Comparison between muscle and BM Ly6C^hi^ cells, *Comparison between muscle Ly6C^hi^ and macrophages (MF), One way ANOVA with Bonferroni multiple comparison post-test **b** Quantitative RT-PCR analysis of ischemic muscle at indicated time points **d**. *n* = 3/4/4/4 mice/group, error bars represent s.e.m., one way ANOVA with Dunnett's Multiple Comparison test. **c** β-galactosidase activity (blue) and CD45 immunohistochemistry (red) in ischemic muscle from *Dll1* ^*+/lacZ*^ reporter mice. Scale bars=50 µm. **d** Representative immunostaining and confocal microscopy of collateral arteries in *Cx3cr1* ^*GFP/+*^ mice after HLI. Phase contrast image (GFP) × 5, scale bar=200 µm. Confocal images with anti-SMA × 40, CD31 × 40 and × 63. Nuclear counterstain (DAPI, blue). Scale bar=20 µm

We also compared expression patterns of Notch target genes in BM monocytes and ischemic monocytes and macrophages. The Notch target genes *Hey1* and *Hes1* were markedly upregulated in cells isolated from ischemic muscle, showing the highest expression levels in macrophages (Fig. 2a). This indicates active Notch signaling during macrophage differentiation in ischemic tissues. At the same time, expression of the Notch ligand Delta-like 1 (*Dll1*) was increased in ischemic muscle with a timecourse matching the appearance of macrophages (Fig. 2b). Analysis of ischemic muscle sections of *Dll1* ^*+/lacZ*^ reporter mice confirmed our previous observation that *Dll1* expression is restricted to arteries (Fig. 2c)^20^. To capture a possible interaction between macrophages and blood vessels we performed HLI in *Cx3cr1* ^*GFP/+*^ reporter mice, followed by in vivo perfusion fixation and confocal microscopy. This revealed adhesion of GFP^+^ cells to the inside of growing collateral arteries at d3 after HLI, the period of highest *Dll1* expression and macrophage induction (Fig. 2d, Supplementary Fig. 3).

**Fig. 3:**
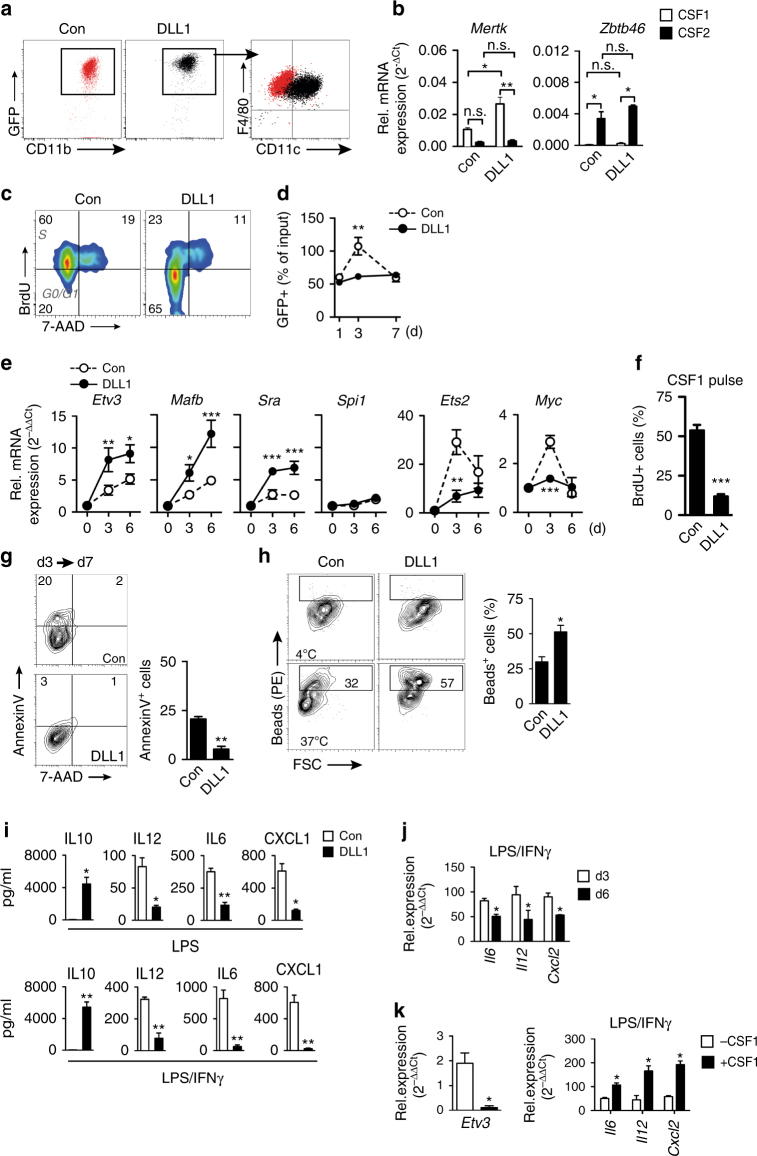
DLL1 instruct macrophage maturation in vitro. **a** Flow cytometry of Ly6C^hi^ monocytes from *Cx3cr1* ^*GFP/+*^ mice cultured for 3d on IgG-Fc (con) or DLL1-Fc chimeric proteins, in the presence of 10 ng/ml CSF1. Representative of *n* = 5 independent experiments. **b** Quantitative RT-PCR analysis at d3 after culture with CSF1, CSF2. *n* = 2 independent experiments performed in duplicates, error bars represent s.e.m. **p < 0.01, *p < 0.05 by one way ANOVA with Bonferroni multiple comparison post-test. **c** Representative analysis of proliferation by 6 h BrdU incorporation at d3. *n* = 3 independent experiments. **d** GFP^+^ cell numbers in culture relative to input number. *n* = 3 independent experiments performed in duplicates, error bars represent s.e.m. **p < 0.01 by two-way ANOVA and Bonferroni multiple comparison post-test. **e** Quantitative RT-PCR analysis normalized to gene expression levels of input Ly6C^hi^ monocytes (d0). *n* = 4 independent experiments performed in duplicates, error bars represent s.e.m. ***p < 0.001, **p < 0.01, *p < 0.05 by two way ANOVA with Bonferroni multiple comparison post-test. **f** BrdU incorporation in CD11b^+^ cells at day 7 after 100 ng/ml CSF1 pulse. *n* = 3 independent experiments, error bars represent s.e.m. ***p < 0.001 by Student’s unpaired *t*-test. **g** Representative Annexin V/7-AAD staining gated on CD11b^+^ macrophages, *n* = 3 independent experiments, error bars represent s.e.m, **p < 0.01, Student’s unpaired *t* test. **h** Representative flow cytometry of phagocytosis (1 h) of PE-labeled dextran beads at indicated temperature, d3 of culture. *n* = 3 independent experiments, error bars represent s.e.m, *p < 0.05, Student’s unpaired *t* test. **i** Multiplex measurement of cytokine concentration in culture supernatant after stimulation with 100 ng/ml LPS (6 h) or combination of 20 ng/ml IFNγ (18 h) and LPS (6 h). *n* = 4 independent experiments, error bars represent s.e.m. **p < 0.01, *p < 0.05, Student’s paired *t* test. **j**, **k** Quantitative RT-PCR analysis after stimulation with IFNγ/LPS normalized to input monocyte levels. Comparison of cells stimulated **j** at d3 or d6, **k** with or without re-stimulation with CSF1 (100 ng/ml) at d6. *n* = 4 independent experiments in duplicates, error bars represent s.e.m. *p < 0.05 by Student’s paired *t*-test

Our in vivo data suggested that Dll1-activated Notch signaling is involved in ischemic macrophage differentiation from monocytes. To test this under defined conditions in vitro we cultured Ly6C^hi^ monocytes from *Cx3cr1* ^*GFP/+*^ reporter mice on recombinant DLL1. Compared with control conditions, DLL1-activated Notch signaling in culture (Supplementary Fig. 4a) and generated CD11b^+^F4/80^+^CD115^+^ macrophages that showed upregulation of GFP, CD11c, and MHC class II, a phenotype resembling ischemic tissue macrophages (Fig. 3a, Supplementary Fig. 4b, c). In contrast, the ligand JAG1 was significantly less efficient in generating this phenotype (Supplementary Fig. 4d). CSF1 was required for these effects, as culture without CSF1 resulted in low-cell yield, while culture of monocytes with CSF2 resulted in complete downregulation of F4/80 and GFP regardless of ligand stimulation (Supplementary Fig. 5a, b). Furthermore, cells cultured on DLL1 in the presence of CSF1 upregulated *Mertk* and expressed CD64 and TLR4 (Fig. 3b, Supplementary Fig. 4e), core macrophage markers, but not the dendritic cell lineage factor *Zbtb46* ^26^, indicating macrophage cell fate. In contrast, culture with CSF2 induced *Zbtb46*, but not *Mertk*, regardless of ligand stimulation (Fig. 3b).

Interestingly, DLL1-generated macrophages mostly remained in the G0/G1 phase of the cell cycle, had suppressed levels of cyclin-dependent kinases *Cdk1* and *Cdk2* and maintained steady cell numbers, while control macrophages showed a high-proliferation rate leading to transient expansion of macrophages in culture (Fig. 3c, d, Supplementary Fig. 4f). Of note, the effects of DLL1 were not explained by changes in surface expression of the CSF1 receptor (CD115, Supplementary Fig. 4b), but were blocked by the γ-secretase inhibitor DAPT, which inhibits Notch receptor activation (Supplementary Fig. 6). Furthermore, DLL1-induced upregulation of macrophage differentiation markers *Mafb* and *Sra1*, but also marked upregulation of the terminal differentiation mediator *Etv3*, and suppression of its downstream repression targets, *Ets2* and *Myc*, while the macrophage master regulator *Spi1* was not influenced (Fig. 3e). Our data thus suggested that DLL1 induces a switch from proliferation to differentiation of macrophages, indicating a role in macrophage maturation. A hallmark of (terminally) differentiated cells is cell cycle arrest in response to growth factor stimulation^8^. To test whether DLL1 promoted terminal macrophage differentiation we re-stimulated cells after 7 days of culture with a CSF1 pulse and quantified the proliferation rate. CSF1 induced proliferation in the majority of control macrophages, while only a small fraction of DLL1-cultured macrophages started to proliferate (Fig. 3f). In addition, DLL1-induced maturation was associated with decreased apoptosis during prolonged culture, contributing to stable cell numbers compared with cell loss observed in immature macrophages (Fig. 3d, g). In functional assays, DLL1 stimulated cells were also found to be more efficient phagocytes (Fig. 3h). Altogether, these data demonstrate a DLL1-induced switch between proliferation and macrophage differentiation.

We next characterized the macrophage inflammatory response. Stimulation of control macrophages with the LPS or LPS/IFN-γ combination, prototypical inflammatory stimuli, induced high secretion of pro-inflammatory cytokines, IL-6, IL-12, and CXCL1, but low levels of the anti-inflammatory cytokine IL-10, which is in line with the current paradigm of macrophage polarization. In contrast, DLL1-generated macrophages secreted much lower levels of pro-inflammatory cytokines, but very high levels of IL-10 (Fig. 3i). These results were confirmed on the level of messenger RNA (mRNA) profiles, which also revealed upregulation of *Arg1* and strong suppression of inducible nitric oxide synthase (*Nos2*), *Cxcl1*, and *Cxcl2* by DLL1 (Supplementary Fig. 7). Thus, DLL1 promotes macrophage maturation, which results in enhanced phagocytic capacity and anti-inflammatory activation even in response to pro-inflammatory stimuli. To test the principle association between maturation and anti-inflammation without the influence of DLL1 we first compared the inflammatory response of early (d3) vs. mature macrophages (d6), as macrophage differentiation progresses over time (Fig. 3d, Supplementary Fig. 8). When stimulated with LPS/IFN-γ, mature macrophages produced less inflammatory cytokines (Fig. 3j). In contrast, pulsing mature macrophages with CSF1 induces downregulation of the differentiation regulator *Etv3*, proliferation, and increased inflammatory responses in response to LPS/IFN-γ (Fig. 3f, k). Altogether, these data demonstrate that macrophage maturation influences the inflammatory response.

### Endothelial cells regulate macrophage differentation in vitro

**Fig. 4:**
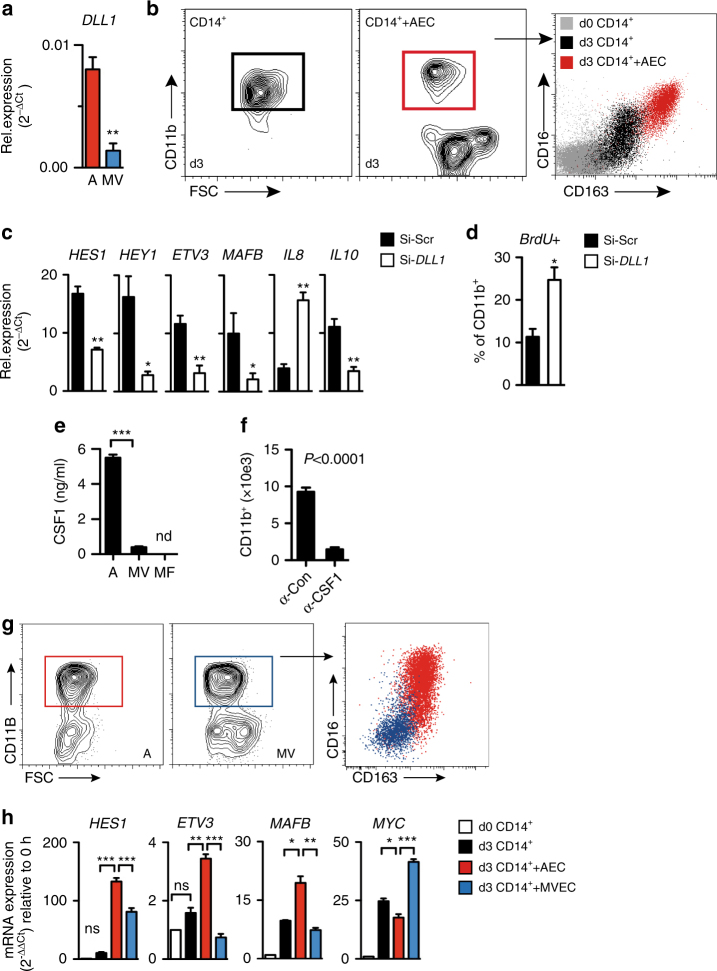
Endothelial Dll1 instructs macrophage maturation in vitro. **a** Quantitative RT-PCR analysis of p3 primary human aortic EC (HAEC, A) or coronary microvascular (MV) EC. *n* = 3 independent samples in duplicates/group, error bars represent s.e.m., **p < 0.01, Student’s unpaired *t*-test. **b** Representative flow cytometry from d3 culture of human CD14^+^ monocytes in the presence of 10 ng/ml M-CSF or HAEC (AEC). *n* = 4 independent experiments. **c**, **d** HAEC were transfected with anti-*DLL1* siRNA (Si-*DLL1*) or scambled control (Scr) before co-culture with CD14^+^ monocytes. **c**. Quantitative RT-PCR analysis of CD11b^+^ isolated from co-culture normalized to expression levels of input monocytes, *n* = 3 independent experiments, error bars represent s.e.m. **p < 0.01, *p < 0.05, Student’s paired *t*-test. **d** BrdU incorporation (6 h) in CD11b^+^ cells from co-culture, *n* = 3 independent experiments performed with duplicate measurements, error bars represent s.e.m. *p < 0.05, Student’s unpaired *t*-test. **e** Protein concentration of CSF1 in co-culture supernatants (A: arterial EC, MV: microvascular EC) or macrophage (MF) supernatants by ELISA, nd: not detectable. *n* = 3 independent experiments performed in triplicates, error bars represent s.e.m. ***p < 0.001 one-way ANOVA with Bonferroni post-test. **f** Number of CD11b^+^ macrophages recovered after co-culture of CD14^+^ monocytes with HAEC treated with isotype (α-Con) and CSF1 neutralizing (α-CSF1) antibody. *n* = 2 independent experiments performed in triplicates, error bars represent s.e.m. *p* < 0.0001, Student’s unpaired *t*-test **g**, **h** Effect of HAEC (A) or MVEC (MV) on CD14^+^ monocytes. **g** Representative flow cytometry and profile of gated population after d3 of co-culture. *n* = 3 independent experiments. **h** Transcriptional profile of CD11b^+^ cells isolated after 3d of culture with EC (A vs MV) or CSF1, normalized to expression levels of input monocytes (d0). *n* = 3 independent experiments, error bars represent s.e.m. ***p < 0.001, **p < 0.01, *p < 0.05, one way ANOVA with Bonferroni multiple comparison test

Our in vivo observations suggested that ischemia generates a transient niche of *Dll1* expressed by arterial EC. Studies with cultured human primary EC from different vascular domains confirmed that arterial EC expressed higher levels of *DLL1* compared with microvascular ECs (Fig. 4a), while levels of Notch ligands *DLL4* and *JAG1* were comparable (Supplementary Fig. 9a). As EC have been shown to promote macrophage polarization^27, 28^, we tested the hypothesis that EC induce macrophage maturation via *DLL1*. We first conducted co-culture experiments of human arterial EC together with human PB CD14^+^ CD16^−^ monocytes, the human equivalent of murine Ly6C^hi^ monocytes^29, 30^, and compared the phenotype to original donor monocytes (d0 CD14^+^) or control culture. At d3, co-cultured CD11b^+^ cells expressed high levels of CD16, CD163, CX_3_CR1, and CD209, a signature consistent with anti-inflammatory macrophages (Fig. 4b, Supplementary Fig. 10)^31^.

To address the role of endothelial Dll1 in macrophage maturation we pretreated EC with a *DLL1*-specific siRNA, which efficiently reduced *DLL1* expression without altering expression of other Notch ligands or Notch target genes (Supplementary Fig. 11a, b). Gene expression analysis of CD11b^+^ macrophages retrieved from co-culture revealed suppressed levels of the Notch-signaling targets *HES1, HEY1*, but also suppression of genes associated with macrophage (terminal) differentiation, *ETV3* and *MAFB*, concomitant with reduced surface expression CD163 (Supplementary Fig. 11c). In addition, expression of *IL10* was reduced, while expression of *IL8* was increased (Fig. 4c). Furthermore, *DLL1* knockdown in EC also lead to a substantial increase in macrophage proliferation, indicating that Dll1 suppresses proliferation and promotes maturation of human macrophages (Fig. 4d).

Arterial EC expressed and secreted significantly more CSF1 than microvascular EC, which in contrast expressed higher levels of CSF2 (Fig. 4e, Supplementary Fig. 9b). Furthermore, secretion of CSF1 was required to generate mature macrophages on arterial EC, as macrophage generation was greatly inhibited by a CSF1-neutralizing antibody (Fig. 4f). We next reasoned that the observed differences in Dll1 expression and myeloid growth factor profiles in the two EC populations should have functional consequences for macrophage instruction. In co-culture experiments comparing arterial vs. microvascular EC, only arterial EC induced differentiation of CD14^+^ monocytes to CD16^+^ CD163^+^ macrophages (Fig. 4g). Furthermore, macrophages generated on arterial EC showed significantly higher levels of *HES1* and *MAFB*, and significantly reduced levels of *MYC*, when compared with either macrophages generated in the presence of microvascular EC or CSF1 (Fig. 4h). Furthermore, the terminal differentiation regulator *ETV3* was only induced in macrophages generated on arterial EC. Altogether, these data demonstrate that human arterial EC have the distinct ability to instruct macrophage differentiation and maturation from monocytes, which requires Dll1.

### EC Dll1 regulates macrophage maturation and arteriogenesis in vivo

**Fig. 5:**
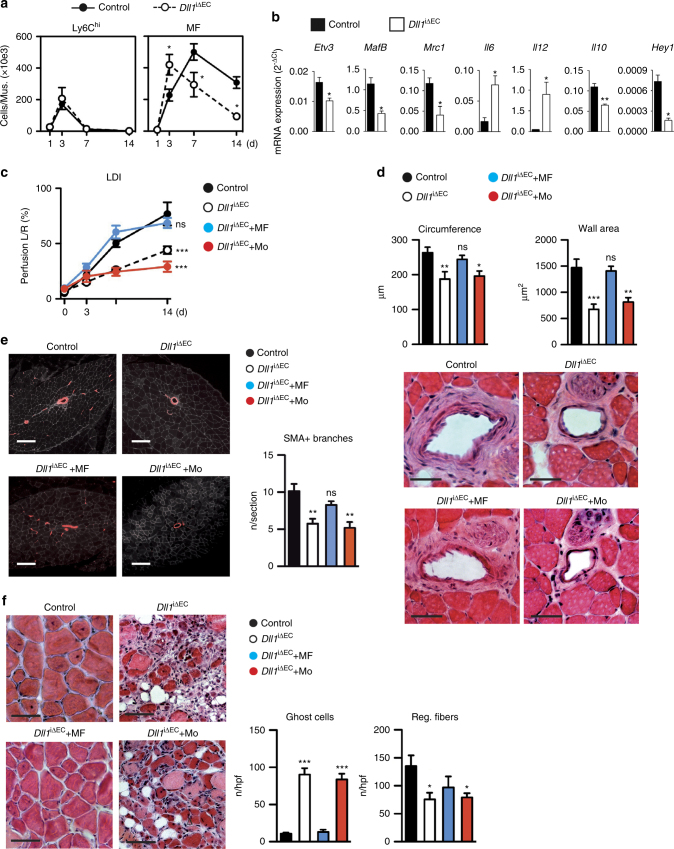
Endothelial Dll1 regulates macrophage maturation and arteriogenesis. **a** Analysis of cell populations by flow cytometry in ischemic muscle of induced endothelial *Dll1* mutant mice (*Dll1* ^*iΔEC*^) and control mice. *n* = 8 mice/group, error bars represent s.e.m. *p < 0.05 by two way ANOVA with Bonferroni multiple comparison test. **b** Gene expression analysis of macrophages sorted from d3 ischemic muscle. *n* = 3 independent experiments, error bars represent s.e.m. **p < 0.01, *p < 0.05, Student’s unpaired *t*-test. **c**–**f** Rescue of endothelial *Dll1* deficiency after HLI by transfer of ischemic macrophages. On d1 after HLI, indicated groups received intramuscular limb injections of 5 × 10e5 macrophages isolated from d3 ischemic muscle (MF) or Ly6C^hi^ monocytes isolated from spleen (Mo) of wt mice, or received control treatment. **c** Foot perfusion by laser Doppler perfusion measurement immediately after induction of HLI (0) and during follow up. *n* = 5/7/7/7 mice/group, error bars represent s.e.m. ***p < 0.001 by two way ANOVA and Bonferroni post test. **d** Quantification of inner circumference and wall area, and representative H&E stained images of collateral arteries d14. Scale bar=50 μm. *n* = 7 mice/group, error bars represent s.e.m. ***p < 0.001 vs control by one way ANOVA and Dunnett’s multiple comparison test. **e** Representative SMA-stained immunofluorescence of muscle sections, Scale bar 100 μm (left) and quantification of arterial branches (SMA^+^) per section d14, *n* = 5 mice/group, error bars represent s.e.m. ***p < 0.001 vs control by one way ANOVA and Dunnett’s multiple comparison test. **f** Representative H&E stained muscle sections. Scale bar=50 μm (left) and quantification of ghost cells and regenerating muscle fibers (right) at d14, *n* = 5 mice/group, error bars represent s.e.m. ***p < 0.001 vs control by one way ANOVA and Dunnett’s multiple comparison test

To test the hypothesis that endothelial Dll1 regulates macrophage maturation after ischemia in vivo we used endothelial-specific and inducible *Dll1* mutant mice (*Dll1* ^*i∆EC*^), which carry conditional *Dll1* alleles and the *Cdh5(PAC)-CreERT2* transgene^21^ and which enable targeting under ischemic conditions (Supplementary Fig. 12a, b). Myeloid cell populations were analyzed by flow cytometry with a non-GFP-based strategy^32^, which gave similar results compared with the GFP-based strategy (Supplementary Fig. 13)^21^. Inducible Dll1 deletion did not change baseline peripheral leukocyte counts (Supplementary Fig. 14a). Following HLI, ischemic muscle recruitment of Ly6C^hi^ monocytes or granulocytes was not significantly altered in conditional Dll1 mutant mice, as were PB leukocyte counts (Fig. 5a, Supplementary Fig. 14b) and expression levels of other Notch ligands (Supplementary Fig. 14c). However, macrophage differentiation kinetics was substantially altered in *Dll1* ^*i∆EC*^ mutant mice, showing increased macrophage numbers initially, followed by a pronounced cell loss later (Fig. 5a). Gene expression analysis from macrophages isolated from ischemic muscle of *Dll1* ^*i∆EC*^ mutant mice revealed significant reductions in macrophage differentiation genes, downregulation of *Il10* and *Hey1*, but upreguation of inflammatory genes *Il6* and *Il12* (Fig. 5b). Thus, endothelial Dll1 regulates macrophage differentiation and inflammatory signature in vivo.

We next addressed the relevance of endothelial Dll1 for arteriogenesis and ischemic muscle recovery^22^. To also address the role of Dll1-dependent macrophage differentiation we designed cell transfer experiments in which either peripheral Ly6C^hi^ monocytes or d3 ischemic muscle macrophages from control donor mice were injected into ischemic limb muscles 1 day after HLI (Supplementary Fig. 15a, b). Hind limb perfusion measurements by laser Doppler imaging showed an impairment in foot perfusion in *Dll1* ^*i∆EC*^ mutant mice, which was improved by transfer of macrophages, but not by transfer of monocytes (Fig. 5c). Histomorphometric analysis of arteriogenesis after HLI showed a significant impairment in luminal and wall growth in collaterals of *Dll1* ^*i∆EC*^ mutant mice, which again was rescued by transfer of macrophages, but not monocytes (Fig. 5d), while *Dll1* ^*i∆EC*^ mutant mice had no baseline collateral phenotype (Supplementary Fig. 16a). As ink perfusion studies also indicated post-ischemic branching defects in *Dll1* ^*i∆EC*^ mutant mice (Supplementary Fig. 16b)^22^ we quantified the number of smooth muscle actin (SMA)^+^ collaterals in ischemic muscle. There was a significant reduction in collateral branch formation in *Dll1* ^*i∆EC*^ mutant mice, which was again rescued by transfer of macrophages, but not monocytes (Fig. 5e). Analysis of capillary density, in contrast, showed an inverse response compared with collateral number, suggesting pronounced ischemia in conditions with reduced collaterals (Supplementary Fig. 16c).

Furthermore, ischemic muscle regeneration was severely impaired in *Dll1* ^*i∆EC*^ mutant mice, indicated by a substantial increase in ghost cells and a significant reduction in regenerating muscle fibers, which was reversed by transfer of macrophages, but not monocytes (Fig. 5f). Taken together, these results suggest that blood vessel restricted Dll1 regulates macrophage maturation and activation state, a function required for arteriogenesis and ischemic tissue regeneration.

### Canonical Notch signaling regulates macrophage maturation

**Fig. 6:**
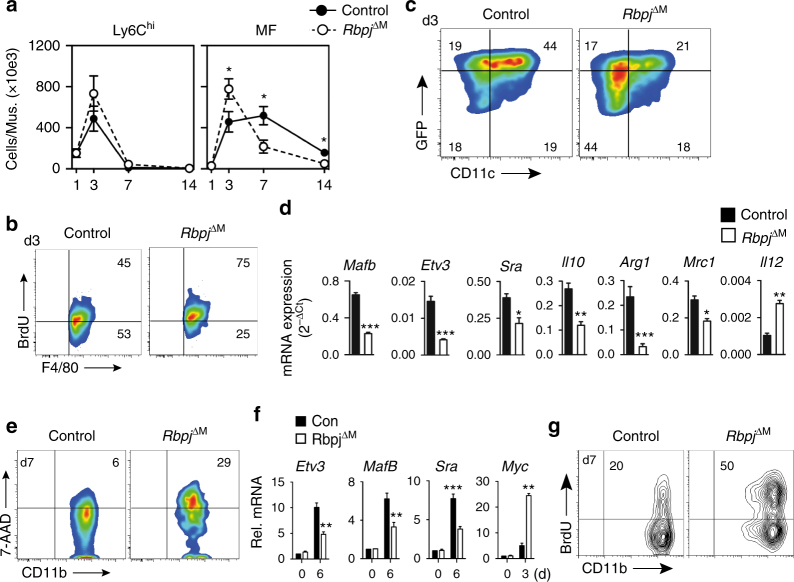
Canonical Notch signaling regulates macrophage maturation in vivo. **a** Analysis of cell populations by flow cytometry in ischemic muscle of *Rbpj* conditional mutant mice (*Rbpj* ^*ΔM*^) and control mice. *n* = 7/9 mice/group, error bars represent s.e.m., *p < 0.05 by two way ANOVA with Bonferroni post test. **b** Representative flow cytometry of 6 h in vivo BrdU incorporation in macrophages from ischemic muscle at d3. *n* = 3 independent experiments **c** Representative flow cytometry of macrophages (CD11b^+^, F4/80^+^) from ischemic muscle at d3. *n* = 4 independent experiments **d** Gene expression analysis of macrophages sorted from d3 ischemic muscle. *n* = 3 independent experiments, error bars represent s.e.m. ***p < 0.001, **p < 0.01, *p < 0.05, unpaired Student’s *t*-test. **e** Representative cell death analysis in macrophages (CD11b^+^, F4/80^+^) from ischemic muscle. *n* = 4 independent experiments. **f** Gene expression analysis of isolated Ly6C^hi^ monocytes from wild type or *Rbpj* ^∆M^ mice (0) and derived macrophages cultured on DLL1 for indicated time. *n* = 3 independent experiments measured in duplicates, error bars represent s.e.m. ***p < 0.001, **p < 0.01, two-way ANOVA and Bonferroni post-test. **g** Representative BrdU incorporation of d7 macrophages on DLL1. *n* = 4 independent experiments

We next asked whether Notch signaling in macrophages regulates ischemic macrophage differentiation and tissue recovery in vivo. We generated mice with conditional ablation of Notch signaling in macrophages (*Rbpj* ^*ΔM*^, Supplementary Fig. 17a) to HLI, which were generated by crossing conditional alleles of the canonical Notch effector *Rbpj* ^33^, a myeloid specific Cre-recombinase, *LysM* ^*Cre*^ ^34^, and the *Cx3cr1* ^*GFP/+*^ reporter. Mice with conditional deletion of *Rbpj* had no alteration in circulating granulocytes or Ly6C^hi^ monocyte numbers at baseline or following HLI, and no alteration in ischemic muscle recruitment (Fig. 6a, Supplementary Fig. 17b). However, differentiation of macrophages was severely altered in conditional *Rbpj* mutant mice, which showed increased numbers of proliferating macrophages (Fig. 6a, b) with impaired upregulation of CD11c and GFP (Fig. 6c). Moreover, gene expression analysis revealed impaired expression of genes involved in macrophage maturation (*Mafb*, *Sra)*, terminal differentiation *(Etv3)*, and antiinflammation (*Mrc1*, *Arg1* and *Il10)*, but upregulation of pro-inflammatory *Il12* (Fig. 6d). Notch-deficiency also resulted in pronounced macrophage loss and increased macrophage cell death at later ischemic timepoints (Fig. 6a, e). Altogether, this phenocopies the kinetics observed in vitro and in conditional *Dll1* mutant mice in vivo.

To corroborate the hypothesis that macrophage maturation from Ly6C^hi^ monocytes requires *Rbpj* we cultured wt or *Rbpj*-deficient Ly6C^hi^ monocytes on DLL1 and compared proliferation and gene expression patterns in developing macrophages. Mutant Ly6C^hi^ monocytes showed no baseline differences in *Etv3*, *Mafb*, *Sra* and *Myc*, and readily gave rise to macrophages in culture (Fig. 6f). However, induction of genes involved in macrophage maturation during culture was severly impaired, and the proliferation rate was significantly increased (Fig. 6f, g), thus demonstrating a requirement for *Rbpj* in macrophage maturation from Ly6C^hi^ monocytes.

**Fig. 7:**
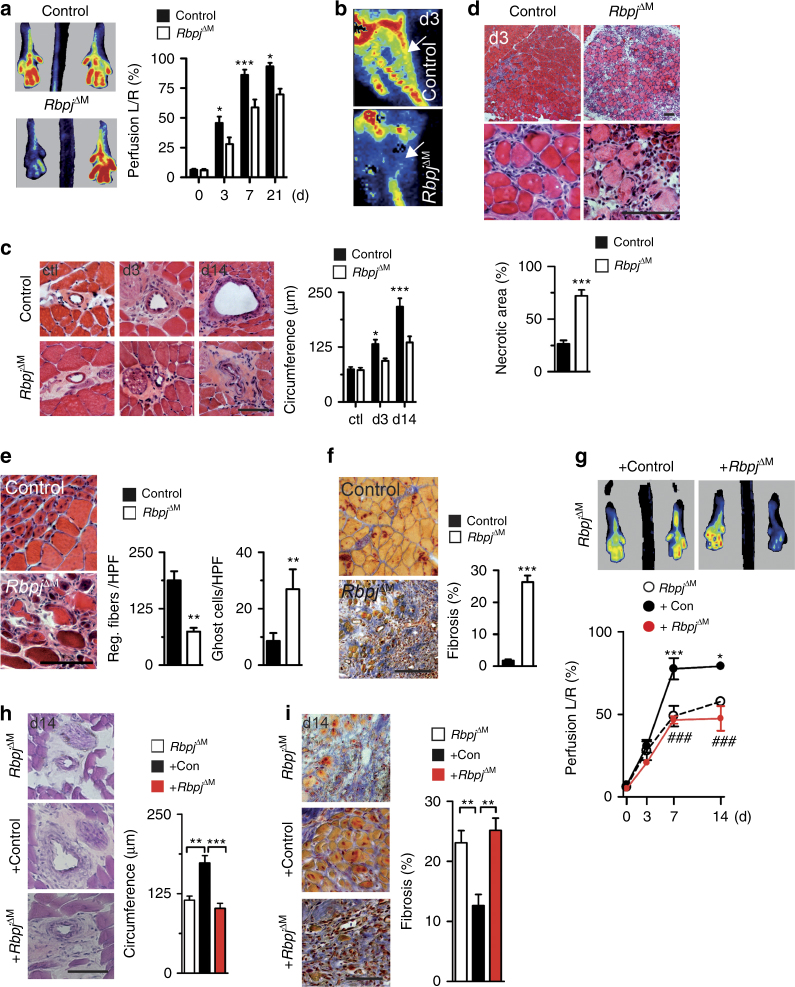
Macrophage Notch signaling regulates arteriogenesis in vivo. **a** Foot perfusion by laser Doppler perfusion measurement after induction of HLI (0) and during follow up. *n* = 8/10 mice/group, error bars represent s.e.m. *p < 0.05, ***p < 0.001 vs control by two way ANOVA and Bonferroni post-test. **b** Representative LDI images of collateral circulation at d3. **c** Representative H&E images of collateral arteries and quantification of inner circumference, *n* = 4/6 (ctl), 9/8 (d3), 7/7 (d14) mice/group, error bars represent s.e.m. *p < 0.05, ***p < 0.001 vs control by two way ANOVA and Bonferroni post-test. Scale bar=100 μm. **d** Overview (×10), magnification (×63) of representative H&E stained muscle sections and quantification of necrosis, *n* = 5 mice/group, error bars represent s.e.m. ***p < 0.001 vs control by Student’s unpaired *t*-test. Scale bar=100 μm. **e** Representative H&E stained muscle sections, quantification of ghost cells and regenerating muscle fibers, *n* = 6/7 mice/group, error bars represent s.e.m. **p < 0.001, Student’s unpaired *t*-test. Scale bar=100 μm. **f** Representative images of Masson-trichrome staining and quantification of fibrosis in muscle sections at d14. *n* = 6/5 mice/group, error bars represent s.e.m. **p < 0.01, Student’s unpaired *t*-test. Scale bar=100 μm. **g**–**i** Adoptive transfer of monocytes on d1 after HLI in *Rbpj* ^*ΔM*^ mice. Intravenous injections of PBS (*Rbpj* ^*ΔM*^) or 3 × 10e5 BM Ly6C^hi^ monocytes from wild type (+Con) or *Rbpj* ^*ΔM*^ mice (+*Rbpj* ^*ΔM*^). **g** Foot perfusion by laser Doppler imaging after induction of HLI (0) and during follow up. *n* = 6/5/5 (d0), 7/5/5 (d3), 6/5/5 (d7), 7/5/5 (d14) mice/group, error bars represent s.e.m. ***p < 0.001, **p < 0.01, *p < 0.05, *comparison between *Rbpj* ^*ΔM*^ and +Con group, # Comparison between +Con and +*Rbpj* ^ΔM^ groups, Two way ANOVA and Bonferroni post test. **h** Representative H&E stained images of collateral arteries at d14 and quantification of collateral circumference, *n* = 7/5/5 mice/group, error bars represent s.e.m. *p < 0.05, ***p < 0.001, one way ANOVA and Bonferroni post-test. Scale bar=100 μm. **i** Representative images of Masson-trichrome staining and quantification of fibrosis in muscle sections at d14, *n* = 7/5/5 mice/group, error bars represent s.e.m. *p < 0.05, ***p < 0.001, one way ANOVA and Bonferroni post-test. Scale bar=100 μm

Laser Doppler perfusion measurements showed impaired foot perfusion (Fig. 7a) and reduced collateral circulation (Fig. 7b). Histomorphometric studies revealed impaired arteriogenesis in *Rbpj* mutant mice after HLI, but showed no baseline phenotype (Fig. 7c). This was accompanied by increased ischemic muscle necrosis (Fig. 7d) and impaired muscle regeneration in conditional *Rbpj* mutant mice (Fig. 7e), which resulted in the development of pronounced muscle fibrosis (Fig. 7f, Supplementary Fig. 17c).

To test whether the ischemic phenotype of *Rbpj*-deficient mice can be rescued by wild type cells we performed HLI in Rbpj^ΔM^ mice and intravenously transferred BM Ly6C^hi^ monocytes of either wild type or Rbpj^ΔM^ mice. Compared with *Rbpj* conditional mutants with control injections, transfer of wild type monocytes improved the perfusion defects (Fig. 7g), arteriogenesis defect (Fig. 7h) and tissue damage (Fig. 7i) following ischemia, while transfer of *Rbpj*-deficient monocytes had no discernible effect.

Overall, these data demonstrate that canonical Notch signaling controls macrophage maturation from monocytes and inflammatory polarization, which is required for arteriogenesis and ischemic tissue repair.

## Discussion

We here define the molecular and cellular context that regulates the origin and function of macrophages in ischemic neovascularization. Taken together, our results show that blood vessels regulate macrophage differentiation from recruited monocytes, which in turn promotes arteriogenesis and ischemic tissue recovery. Macrophage maturation is controlled by Notch ligand *Dll1* expressed in vascular endothelial cells and requires macrophage canonical Notch signaling via *Rbpj*. Thus, blood vessels foster macrophages for arteriogenesis in response to ischemia.

The origin and maintenance of macrophages after injury can occur either through their differentiation from recruited Ly6C^hi^ monocytes, via local macrophage proliferation or migration of mature macrophages. Using monocyte cell fate tracking and timed elimination studies we show that the transient population of macrophages that arises in ischemic tissues displays a distinct phenotype and develops from an early invading wave of Ly6C^hi^ monocytes by way of maturation. This not only specifies the role of monocytes and macrophages in arteriogenesis^4, 5^, but also confirms and extends previous reports that Ly6C^hi^ monocytes give rise to macrophages during myocardial ischemia and healing^11^.

The angiocrine functions of blood vessels, which occur independent of blood flow, are involved in organ maintenance and regeneration^12^. After injury, instructive cues expressed by local endothelial cells orchestrate the injury response of tissue-resident progenitor cells or fibroblasts during organ regeneration, which in part is mediated by endothelial-specific expression of Notch ligands such as *Jag1* ^13–15^. We had previously shown that *Dll1*, which is upregulated in arterial EC upon ischemia, is an essential regulator of arteriogenesis and tissue regeneration^20^. Our finding that endothelial *Dll1* regulates macrophage maturation in vitro and in vivo, which is required for arteriogenesis and ischemic tissue regeneration, extends the spectrum of angiocrine functions towards the setting of ischemic injury, but also demonstrates that angiocrine signaling via Notch ligand *Dll1* supports tissue recovery by instructing maturation of recruited cell populations. This is further supported by our finding that transfer of ‘niche’ educated macrophages improves arteriogenesis and tissue repair after HLI. Furthermore, these data also clearly demonstrate a specific, non-redundant role of vascular *Dll1* in regulating macrophage maturation, which again emphasizes the ligand-specific nature of Notch signaling events^19^. Our data also support and expand previous reports on how EC populations cause macrophage polarization^28^. However, while mature macrophages seem to mediate most of the downstream vascular effects of *Dll1*, our findings do not necessarily exclude the possibility of *Dll1*-dependent, intermediary processes occurring within blood vessels involved in the ischemic response.

In addition, our findings also support and extend the concept of on-site education of VEGF-recruited monocytes for arteriogenesis, in which Notch has been implicated^35^. Altogether, our data clearly demonstrate that angiocrine signaling contributes to neovascularization and tissue recovery via macrophage maturation, thus extending the spectrum of endothelial function. On the other hand, it is possible that dysregulated angiocrine functions are involved in endothelial dysfunction, which is thought to mediate the progression of chronic vascular disease^36^.

Macrophage diversity is considered to arise from a combination of differentiation programs and activation programs in response to external stimuli^6, 9, 10^. Our data contribute to the current model in several ways. We demonstrate a requirement for canonical Notch signaling, activated by vascular *Dll1*, for macrophage differentiation and maturation from Ly6C^hi^ monocytes, thus identifying an angiocrine identity factor for macrophage differentiation. Active Notch signaling promoted a mature phenotype with enhanced phagocytic activity, a low proliferation rate in response to CSF1, improved survival and induction of the terminal differentiation regulator Etv3, thus demonstrating multiple aspects of mature macrophages^8^. Of note, a related function of Notch in promoting myeloid differentiation has recently been described in myeloid leukemia^37^. At the same time, macrophage maturation induced by Notch generated cells with high-phagocytic abilities, demonstrated in vitro and suggested by fewer ghost cells in vivo, which also showed an activation profile consistent with tissue-resident or M2-like macrophages^7, 38^. On the other hand, we also demonstrate that activation/polarization of macrophages and their inflammatory potential is a function of macrophage maturation. Activation of mature macrophages instructed by Notch signaling promoted an anti-inflammatory response under conditions that induce pro-inflammatory cytokines in control macrophages. In addition, the evolution of macrophage maturation over time also dampened the inflammatory response, while re-stimulation with CSF1 induced proliferation and promoted inflammation. Furthermore, the association between macrophage maturation and inflammatory response was also observed in vivo. This demonstrates that maturation critically influences polarization and suggests a modified perspective on macrophage diversity. This hypothesis is also supported by recent findings from systematic gene expression profiling of monocyte to macrophage differentiation, which demonstrated that compared with monocytes, naive macrophages display attenuated innate inflammatory pathways, most likely necessary to generate functional tissue macrophages^39^.

Our study, in which ligand, cell-source and growth factor context were specifically defined, clearly demonstrates on several levels an anti-inflammatory role of canonical Notch signaling induced by *Dll1*, which confirms and extends previous reports^40, 41^. However, other studies show a pro-inflammatory role for Dll4/Notch signaling^42–44^. This suggests that the role of Notch is ligand-, cell-, and context-specific, which again emphasizes the differential effects of specific ligand-receptor combinations^19^. Nevertheless, together the studies now provide a framework to study the role of Notch signaling components for macrophage phenotypes in specific contexts. Currently, the molecular mediators of Notch in macrophage differentiation and function are unclear. Importantly, loss of *Phd2*, the enzyme controlling HIF-1α activity, has also been shown to induce macrophage polarization and arteriogenesis^5^. This would potentially provide a possible mechanism, since Notch and HIF-1α interact under hypoxic conditions in precursor cells during development^45^. The framework described here can also help to define the factors by which macrophages promote arteriogenesis. Further studies clearly are required to address these issues.

Furthermore, the regulation of cell fate choices of Ly6C^hi^ monocytes are only beginning to emerge. Our cell fate tracking studies of Ly6C^hi^ monocytes during ischemia revealed that, in ischemic muscle, monocytes adopt a macrophage cell fate, but that in other tissues, such as the spleen, cells convert to Ly6C^lo^ monocytes, a process which also requires endothelial Dll1^21^. Thus, while endothelial Dll1 clearly is required for both processes, our findings also suggest that additional, so far unknown, signals ultimately determine cell fate of Ly6C^hi^ monocytes. These signals might constitute tissue-specific factors, or pathology-related milieus, or both.

Last but not least, we here describe a cell culture technique for generation of terminally differentiated macrophages from monocytes under defined conditions ex vivo. These macrophages feature a stable anti-inflammatory phenotype even when challenged with pro-inflammatory stimuli. This provides a setting to study and understand the molecular events driving macrophage maturation and diversity under defined conditions. Moreover, since a recent trial in patients with severe heart failure showed positive results with cell therapy employing an ex vivo expanded cell product enriched for anti-inflammatory ‘M2’ macrophages^46^, our description might also prove useful to refine cell-therapeutic applications of ex vivo generated macrophages to improve chronic ischemia.

## Methods

### Mice

Experiments were approved by the State Government (LAVES, Lower Saxony). Mouse strains (Supplementary Table 2) were housed under specific pathogen-free conditions. Around 10–12 week old male mice were used with age- and sex-matched littermate controls.

### Mouse strain information

*Cx3cr1* ^*GFP/+*^ mice^23^, *Dll1* ^*+/LacZ*^ mice^47^, *LysM* ^Cre^ mice^34^, *Rbpj* ^lox/lox^ mice^33^, *Dll1* ^lox/lox^ mice^48^, *Cdh5(PAC)-CreERT2* mice^19^ have been described. *Gt(ROSA)26Sor* (Stock number:003309) mice carrying Cre-inducible *lacZ* alleles were obtained from The Jackson Laboratories and *B6.SJL-Ptprc* ^*a*^ *Pepc* ^*b*^ */BoyJ* (CD45.1^+^) (Stock number:002014) mice were obtained from Charles River. The description of the genetic strains used in the study is described in Supplementary Table 2.

### Hind limb ischemia

Hind limb Ischemia (HLI), laser Doppler imaging, tissue preparation and analysis of arteriogenesis and muscle sections was carried out as described^22^ with the following modification: mice were anaesthetized with a mixture of Ketamine (80 mg/kg, Pfizer), Xylazine (2.5 mg/kg, Bayer healthcare), and Midazolam (2.5 mg/kg, Hexal). Briefly, the superficial branch of the left femoral artery was surgically ligated distal to the origin of the deep femoral branch, and growth of pre-existing collateral arteries supplied by the deep femoral branch was studied in semimembranosus muscle sections. Perfusion was measured by Laser Doppler Imaging of plantar regions of interests with Perimed LDPI PIM II Laser Scanner (Perimed, Sweden) and calculated as ratio of left vs. right values. For flow cytometry cell preparation the anterior tibial muscles were anatomically excised and digested in DMEM containing 2 mg/ml collagenase II (type 2, derived from *Clostridium histolyticum*, Catalog no#LS004176, Worthington Biochemical Corp.) at 37 °C for 20 min using Gentle MACS dissociator (Miltenyi) and filtered through a nylon mesh (40 µm), after which total viable cell number was determined with Trypan blue in a Neubauer chamber.

### Animal treatment

Anti-CCR2 mAb (MC21)^24^ or rat IgG2b control antibody was administered i.p. once a day at a dose of 22.5 µg/mouse, at day 0–2, 0–4, or 4–6 post HLI. Tamoxifen-regulated Cre-recombinase activation was induced as described^21^ two weeks before experiments. BrdU (1.5 mg, BD Biosciences) was injected i.p. and incorporation was detected after 6 h using BrdU flow kit (BD Biosciences).

### Adoptive transfer experiments

Approximately 1–1.5×10^6^ CD11b^+^GFP^+^Ly6C^hi^ monocytes were sorted from the bone marrow of CD45.2 *Cx3cr1* ^*GFP/+*^ mice and injected i.v. into CD45.1 mice 1 day after HLI for cell fate tracking studies. For ischemic rescue experiments cells were administered i.v. or through intramuscular injection where indicated. Lin^neg^CD11b^+^Ly6C^hi^ F4/80/I-A^b^/CD11c^lo^/^neg^ splenic monocytes or Lin^neg^CD11b^+^ F4/80/I-A^b^/CD11c^+^ macrophages (Supplementary Table 1) sorted from d3 ischemic muscle were injected through intramuscular injection into ischemic recipient muscles at d1 after HLI at a dose of 5 × 10^5^ cells in PBS. In a separate rescue experiment 3 × 10^6^ CD11b^+^GFP^+^Ly6C^hi^ monocytes were administered through i.v. injection.

### Tissue histology and immunohistochemistry

Immunohistochemistry, β-galactosidase, and immunofluorescence staining in mice were performed with modifications from previous descriptions^20–22^. Mice were killed and perfused with 0.1% adenosine (Sigma)/0.05% BSA (Roth)/5000 U heparin (Ratiopharm), followed by fixative 4% PFA (Sigma). Muscles were excised, incubated in 15% (6 h) and 30% (18 h) sucrose and embedded in Tissue-tek OCT compound (Sakura). β-galactosidase staining was performed on PFA fixed slides at 37 °C. Slides were co-stained with anti-mouse CD45 and appropriate alkaline-phosphatase-labeled secondary antibody, then counterstained with eosin, mounted in Vitro-Clud^®^ medium (R. Langenbrinck Labortechnik) and analyzed with Olympus IX71 microscope. For immunofluorescence (IF) and confocal laser scanning microscopy (CLSM) tissue sections were stained using anti-SMA, anti-CD31, and appropriate fluorescence-conjugated secondary antibodies, DAPI and slides were mounted in fluorescence mounting medium (DAKO). Detailed information of the antibodies used can be found in Supplementary Table 5. Whole-mount arterial imaging in limb muscles was performed with a 160 mg/ml pigment particle solution (Schmincke, cat. no. HKS 318) infused via the left ventricle. Images were acquired using Leica TCS SP2 AOBS (Leica Microsystems, Germany) confocal microscope or Zeiss Observer Z1 fluorescence microscope (Zeiss, Germany) respectively.

### Flow cytometry

Single-cell suspensions from tissue homogenates or in vitro culture were treated with anti-mouse or anti-human CD16/32 (Trustain-fcX, Biolegend for mouse and FcR Blocking Reagent, Miltenyi Biotec for human) to prevent non-specific binding of antibodies to Fc-receptors. To evaluate apoptosis, cells were stained with 7-AAD and AnnexinV in AnnexinV-binding buffer (all from BioLegend), according to manufacturer’s protocol. After washing, cells were re-suspended in PBS containing 2% FCS, 2 mM Na_2_EDTA and labeled with primary and secondary antibodies or streptavidin-fluorochrome conjugates (Supplementary Table 5). After staining, cells were washed twice and stained with 7-AAD or Propidium Iodide (Sigma) to exclude dead cells in analysis. The cell suspension was then analyzed on a FACSCalibur or LSR II (BD Biosciences). Sorting was performed on FACSAria (BD Bioscienses) and XDP (Beckman Coulter).

For determination of cell numbers per muscle, the complete anterior tibial muscle was anatomically excised, digested, and total live cell number determined in a Neubauer chamber with trypan blue staining. After flow cytometry, the number of cells per population was determined with the following formula: Trypan blue^neg^ cells×frequency of live/100.

### Phagocytosis assay

Macrophages were washed, resuspended in RPMI with 10% FCS, and 10^5^ cells were incubated with 1 μm fluoresbrite red latex beads (Polysciences) at a ratio of 10 beads per cell^49^. The tubes were incubated at 37 °C or on ice for 1 h, washed and uptake of fluorescent beads was analyzed by flow cytometry.

### Quantification of cytokines and chemokines

Approximately 50 µL of cell free supernatants from in vitro cultured macrophages were mixed with 50 µL of the Bioplex resuspension buffer and concentration of cytokines and chemokines was determined using Bio-Plex^®^ Mouse cytokine kit (Biorad) on a Luminex 200 according to manufacturer’s instructions.

### Murine cell culture and human co-culture experiments

CD11b^+^GFP^+^Ly-6C^hi^ monocytes were sorted from the bone marrow of *Cx3cr1* ^*GFP/+*^ mice and cultured in RPMI1640 supplemented with 10% FCS and either 10 ng/ml recombinant murine CSF1 or CSF2 (Peprotech) based on the experiment. Ligand coating was performed with 1 µg/ml of DLL1-Fc and JAG1-Fc chimeric proteins where indicated (R&D Systems) reconstituted in PBS containing 0.1% BSA for 6 h at room temperature. Recombinant mouse IgG2A-Fc was used as a control. For Notch inhibition assays cells were treated with DAPT (N-[N-(3,5-Difluorophenacetyl)-L-alanyl]-S-phenylglycine t-butyl ester, 3 µM/ml, Sigma Aldrich, Germany), following cell seeding on ligands, and was added to the culture every 24 h. DMSO (Sigma Aldrich, Germany) was added to culture as a control.

Human aortic EC (HAEC, Cat no.: CC-2535), cardiac microvascular EC (MV, Cat no.: CC-7030) were expanded in EGM-2 and EGM-2MV bullet kit medium, as recommended by manufacturer (Clonetics Endothelial cell system, Lonza) and used at P3. Human PB monocytes were isolated with CD14 microbeads (Miltenyi Biotec) and cultured with EC or human M-CSF (Peprotech, 10 ng/ml) in medium supplemented with 1% FCS. For co-culture experiments, EC were seeded at 5000 cells/cm^2^ and grown for 2 d to form a confluent monolayer. 4 × 10^4^ cells/cm^2^ CD14^+^ monocytes were seeded and co-cultured for 24 or 72 h in EBM2 supplemented with 1% FCS+Gentamycin/Amphotericin. Re-isolation after co-culture was performed with CD11b microbeads (Miltenyi Biotec). For estimation of protein levels of CSF1 in co-cultures, 100 µl of the sample aliquots was used for CSF1 estimation in a solid phase sandwich ELISA (96 well format, R&D systems) as per manufacturer’s instructions and the optical density was read at 540 nm. For human CSF1 neutralization experiment, co-cultures were treated with 2 μg/ml anti–CSF1 mAb (Catalog no #MAB216) or isotype mAb (mouse monoclonal IgG_2A_) from R&D biosciences.

### siRNA knockdown experiments

For siRNA studies, cells were transfected with Silencer® Pre-designed siRNA DLL1 (100 nM) (siRNA ID: 133774, Ambion, Applied Biosystems) using GeneTrans II transfection reagent MoBiTec GmbH). Scrambled RNA was used as control. Co-culture of monocytes was established 48 h after transfection.

### RNA analysis by real-time quantitative PCR

RNA was isolated with the Nucleospin II kit (Macherey Nagel) and reverse transcription was done using Reverse transcriptase Kit (Invitrogen), according to manufacturer’s protocols. For gene expression analysis, primers were designed using the software Primerquest (IDT) and PrimerBLAST (NCBI) following general guidelines for primer design. Quantitative RT-PCR analysis was performed in duplicates for each sample using Fast start essential green DNA master mix (Roche GmbH) according to manufacturer’s instructions with primers listed in Supplementary Tables 3 and 4 on Light cycler 96 system (Roche). Murine/Human *Rps9* was used as a housekeeping gene to normalize expression of gene of interest depending on the experiment. Relative expression was calculated by the comparative CT (2^−ΔΔCt^ values indicate fold change of the gene of interest in the samples relative to a selected control, 2^−ΔCt^ values indicate relative expression of gene of interest between samples normalized to *Rps9*)^50^.

### Statistical analysis

Results are expressed as mean ± s.e.m. *n* numbers indicate biological replicates of experiments performed at least three times unless otherwise indicated. Comparison between two groups was calculated using unpaired, two-tailed Student’s *t*-test or two-tailed paired Student’s *t*-test with confidence interval of 95% as indicated. For comparison of multiple experimental groups either one-way ANOVA or two-way ANOVA was performed where indicated. Dunnett’s multiple comparision posttest or Bonferroni’s post-test was performed where indicated after performing multiple comparison with ANOVA when the overall *p* value was  < 0.05.

### Data availability

The authors declare that all the relevant data are available upon request.

## Electronic supplementary material

Supplementary Information
