# Supplementary material for: Blood vessel control of macrophage maturation promotes arteriogenesis in ischemia
Source: Nat Commun. 2017 Oct 16;8:952. doi: 10.1038/s41467-017-00953-2 (PMC5643305; doi:10.1038/s41467-017-00953-2)
Supplement: Supplementary file 1 — Supplementary Information [file 41467_2017_953_MOESM1_ESM.pdf]

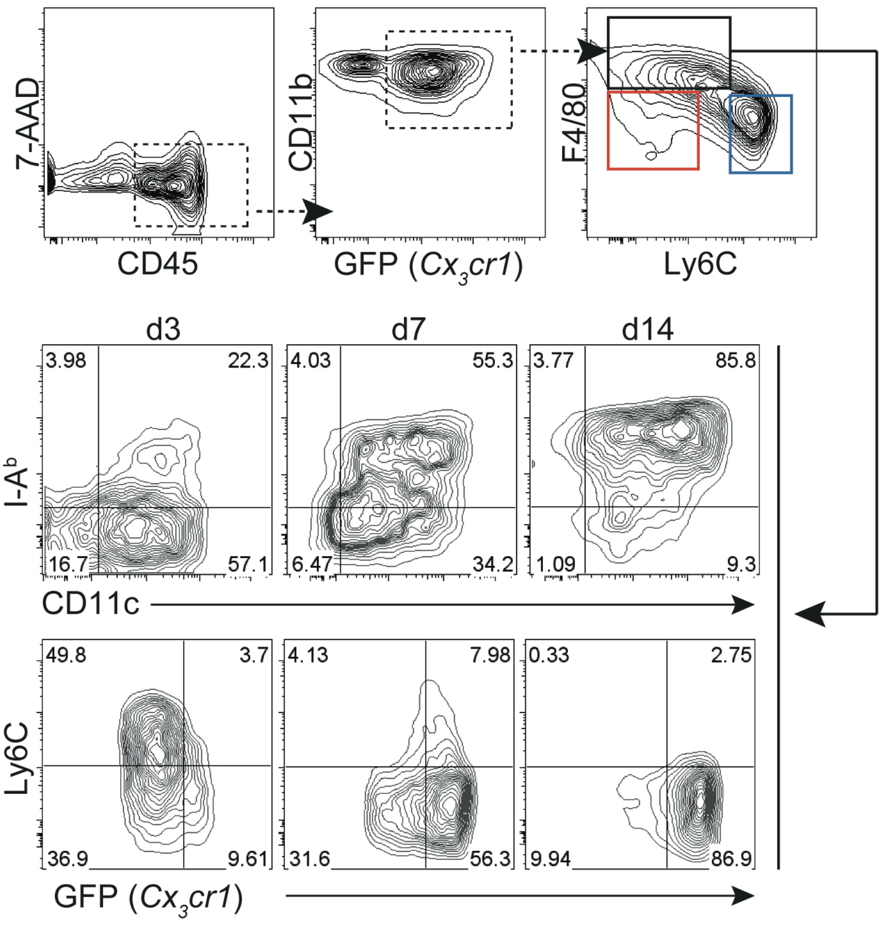

Supplementary Figure 1: Gating strategy and quantification of myeloid cells in ischemic muscle of *Cx3cr1*$^{GFP/+}$ mice post HLI.

Representative flow cytometric analysis of ischemic *tibialis anterior* muscle of *Cx3cr1*$^{GFP/+}$ mice. Macrophages (F4/80^{+}Ly6C^{-}, black), Ly6C^{hi} (blue), Ly6C^{lo} monocytes (red), n=3 independent experiments

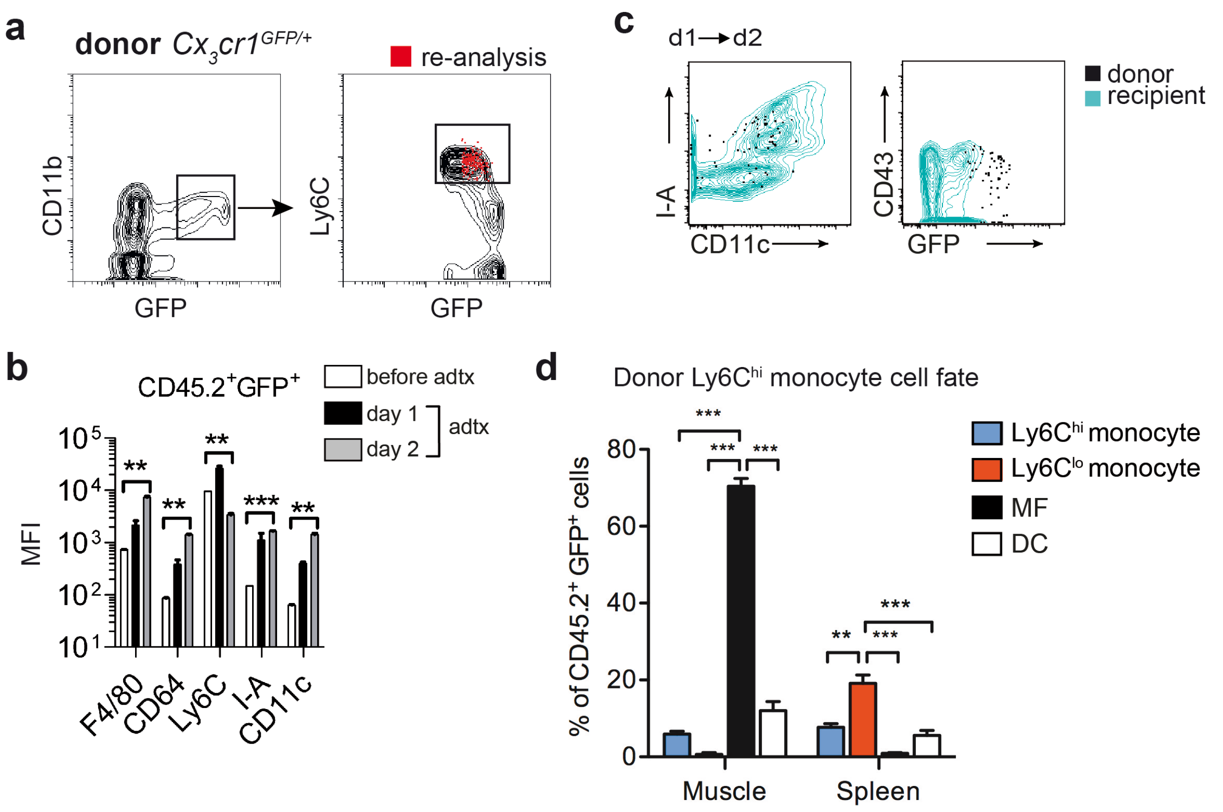

Supplementary Figure 2: Adoptive transfer of Ly6C^{hi} monocytes after HLI. (a) Sorting strategy for Ly6C^{hi} monocytes from bone marrow of *Cx3cr1$^{GFP/+}$* donor mice. The sorted cells (red) were re-analyzed for purity and overlayed to initial sort file. **(b)** Change in mean fluorescent intensity (MFI) of surface markers on donor CD45.2^{+}GFP^{+}. Data cumulative of n=3 independent experiments, error bars represent s.e.m.**p<0.01, Student's unpaired t-test between naive (adtx) and d2 transferred cells. **(c)** Phenotype of donor cells in recipient muscle, representative of n=2 independent experiments. **(d)** Cell fate analysis at d2 of donor CD45.2^{+}GFP^{+} monocytes recovered from ischemic muscle and spleen. n=3 independent experiments, error bars represent s.e.m. ***p<0.001, **p<0.01, Two-way ANOVA with Bonferroni post-test.

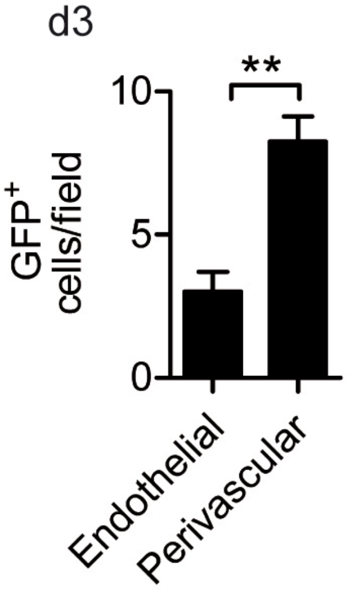

Supplementary Figure 3: GFP$^+$ cell localization in collateral arteries in *Cx3cr1*$^{GFP/+}$ mice after HLI.

(a) Quantification of GFP$^+$ cells/field near collateral artery at d3. n=4 mice, error bars represent s.e.m. **$p<0.01$, Student's unpaired t test.

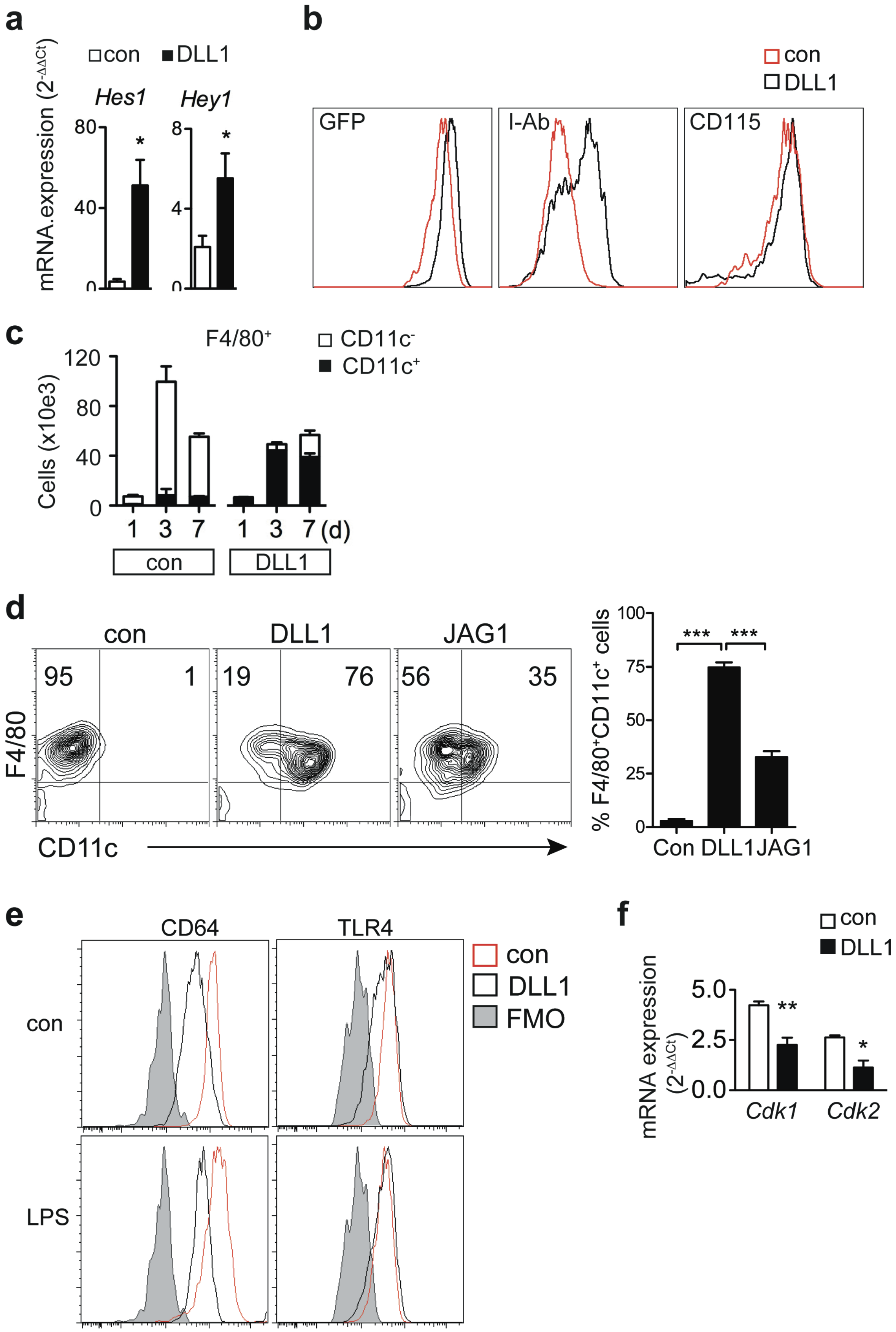

a
con
DLL1
mRNA.expression (2-ΔΔCt)
Hes1
Hey1
80
40
0
8
4
0
*
*
b
con
DLL1
GFP
I-Ab
CD115
c
F4/80+
CD11c-
CD11c+
Cells (x10e3)
120
80
40
0
1 3 7 1 3 7 (d)
con
DLL1
d
con
DLL1
JAG1
95
1
19
76
56
35
F4/80
CD11c
% F4/80+CD11c+ cells
100
75
50
25
0
***
***
Con DLL1 JAG1
e
CD64
TLR4
con
DLL1
FMO
con
LPS
f
con
DLL1
mRNA expression (2-ΔΔCt)
5.0
2.5
0.0
**
*
Cdk1
Cdk2

Supplementary Figure 4. Transcriptional and phenotypic changes induced by DLL1.

Ly6C^{hi} monocytes were cultured in plates coated with DLL1 or control. **(a)** Quantitative RT-PCR analysis at d3 normalized to gene expression levels of input Ly6C^{hi} monocytes. n=3 independent experiments performed in duplicates, error bars represent s.e.m. *p <0.05, Student's paired t-test. **(b)** Representative histograms depicting expression of surface markers at d3. Image is a representative of n=2 independent experiments. **(c)** Quantification of absolute numbers of CD11b^{+}GFP^{+} macrophages analysed for F4/80^{+}CD11c^{+} expression at d1,3 and 7 of culture, error bars represent s.e.m. Data is representative of n=4 independent experiments performed in duplicates. **(d)** Flow cytometry of Ly6C^{hi} monocytes from *Cx3cr1$^{GFP/+}$* mice cultured for 3d on IgG-Fc (con), DLL1-Fc and JAG1-Fc chimeric proteins, in the presence of 10ng/mL CSF1. n=3 independent experiments, error bars represent s.e.m, ***p<0.001, one-way ANOVA with Bonferroni post-test. **(e)** Flow cytometry of cells cultured on control (IgG2A-Fc) or DLL1-Fc for 6d in presence of murine CSF1. FMO was used as staining controls. Data show representative histograms of n=3 independent experiments. **(f)** Expression of Cdk1 and Cdk2 by quantitative RT-PCR at d3 normalized to gene expression levels of input Ly6C^{hi} monocytes (d0). n=3 independent experiments, performed in duplicates, error bars represent s.e.m. **p<0.01 and *p <0.05, Student's paired t-test.

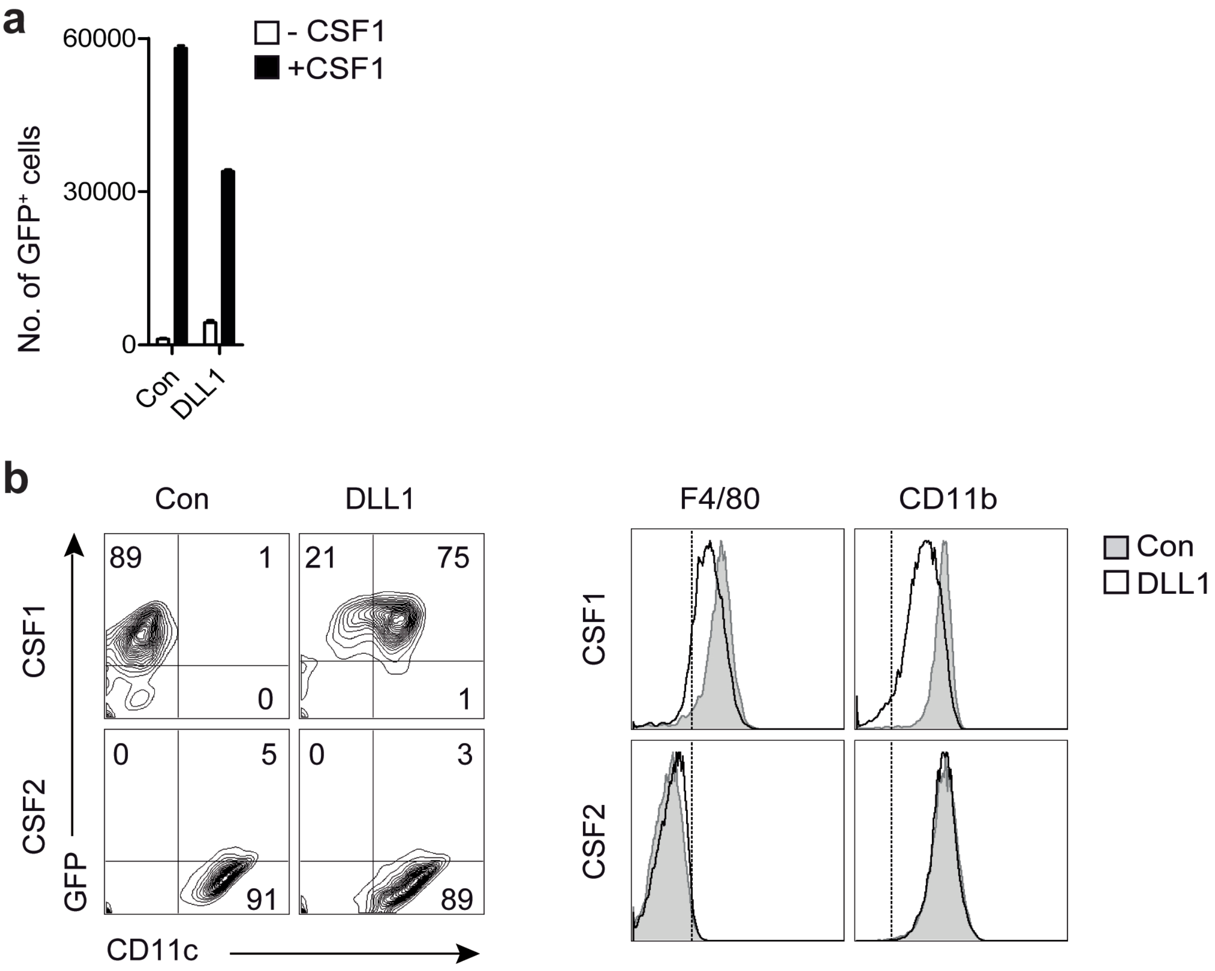

Supplementary Figure 5: Effects of CSF1 and CSF2 on macrophage phenotypes

Ly6C^{hi} monocytes were cultured in plates coated with DLL1 or control (IgG2A-Fc). **(a)** Quantification of absolute numbers of CD11b^{+}GFP^{+} macrophages at d3 cultured in the presence (+CSF1) and without (-CSF1) from the day of seeding. Data is representative of n=2 independent experiements performed in duplicates, error bars represent s.e.m. **(b)** Ly6C^{hi} monocytes were cultured with CSF1 or CSF2 at 10ng/ml and analyzed for the expression of GFP, CD11b, CD11c and F4/80 at d3. Data show representative blots and histograms of flow cytometry, n=3 independent experiments.

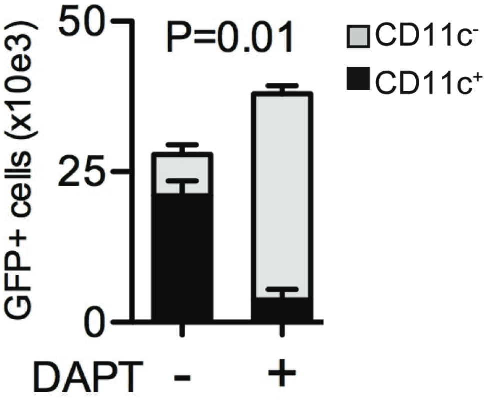

Supplementary Figure 6: Effect of Notch inhibition by DAPT on macrophage numbers and phenotype.

Ly6C^{hi} monocytes were cultured on DLL1 in the presence of either γ-secretase inhibitor DAPT (+) or carrier DMSO (-). Cells were gated for GFP and CD11c expression and absolute numbers in gate were quantified. n=3 independent experiments, error bars represent s.e.m., comparison for CD11c^{+} population by Student's paired t-test.

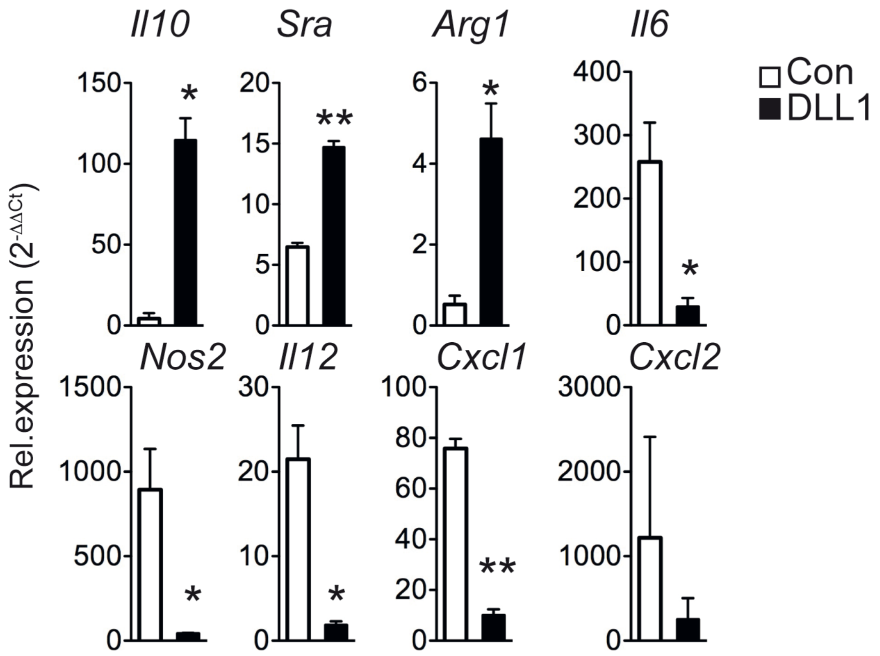

Supplementary Figure 7: Effect of DLL1 on macrophage activation profile after stimulation with IFNγ/LPS.

Ly6C[hi] monocytes were cultured in plates coated with DLL1 or control (IgG2A-Fc), in presence of 10ng/ml murine CSF1. At day 5, medium was exchanged and cells were stimulated with 20ng/mL IFN-γ for 12hours and followed by LPS stimulation for 6 hours (final concentration of 100ng/mL). Transcriptional profiling of genes was performed with quantitative RT-PCR normalized to gene expression levels of unstimulated d6 macrophage. Data is cumulative pool of n=3 experiments performed in duplicates, error bars represent s.e.m. **p<0.01, * p<0.05, students paired t-test.

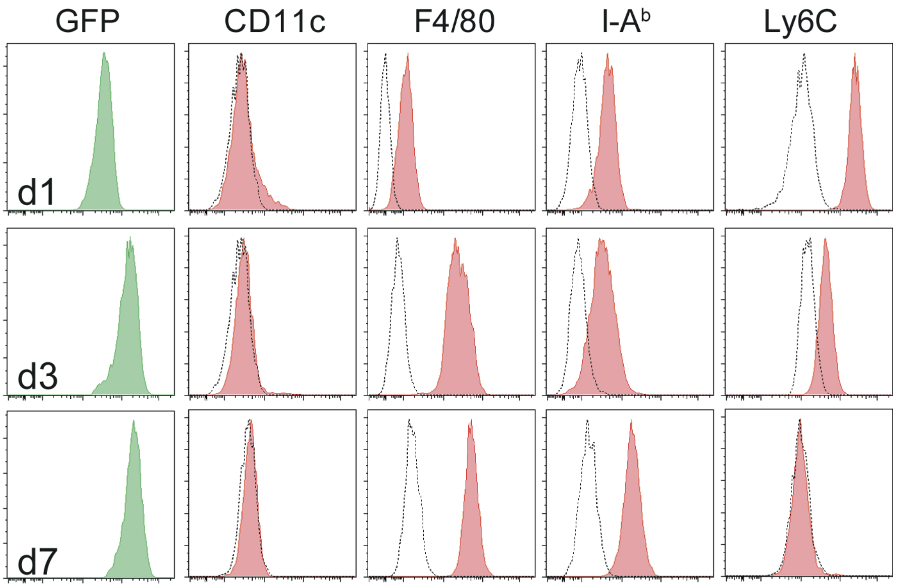

Supplementary Figure 8: Dynamics of surface marker expression during differentiation of monocytes to macrophages.

Ly6C^{hi} monocytes were cultured in plates coated with control (IgG2A-Fc), in presence of murine CSF. Expression of surface markers was determined at days 1, 3 and 7 of culture. Data show representative flow cytometric analysis of n=3 independent experiments.

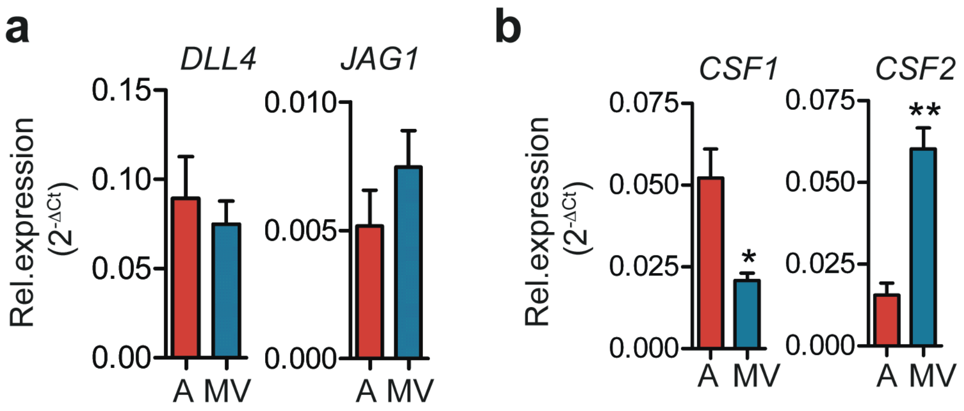

Supplementary Figure 9: Comparision of arterial vs. microvascular EC.
(a) Quantitative RT-PCR analysis of p3 primary human aortic EC (HAEC, A) or coronary microvascular (MV) EC. n=3 independent samples in duplicates/group, error bars represent s.e.m. **(b)** Quantitative RT-PCR analysis of co-cultured EC, isolated by $CD31^{+}/CD11b^{-}$ sorting. Data is cumulative of n=3 independent experiments, performed in duplicates, error bars represent s.e.m. *$p<0.05$, **$p<0.01$, student's unpaired t-test.

a

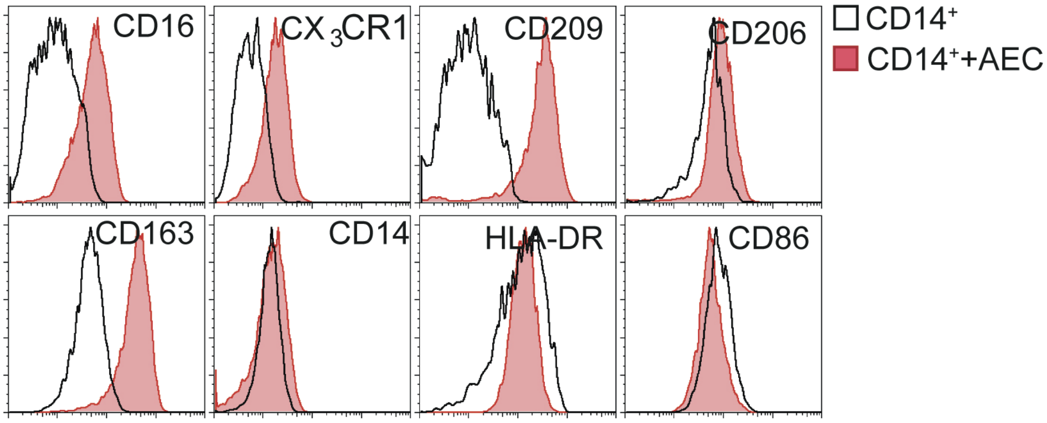

Supplementary Figure 10: Analysis of CD14$^+$ monocytes co-cultured with arterial endothelial cells (AEC)

(a) HAEC and CD14$^+$ monocytes were co-cultured for 3 days. Co-cultured cells were gated for CD11b expression and analyzed by flow cytometry. Figure is a representative histogram comparing expression of macrophage surface markers on CD14$^+$ cultured with M-CSF (open line) and CD14$^+$ co-cultured with arterial endothelial cells (AEC, red tinted). Data is representative of n=3 experiments performed in duplicates.

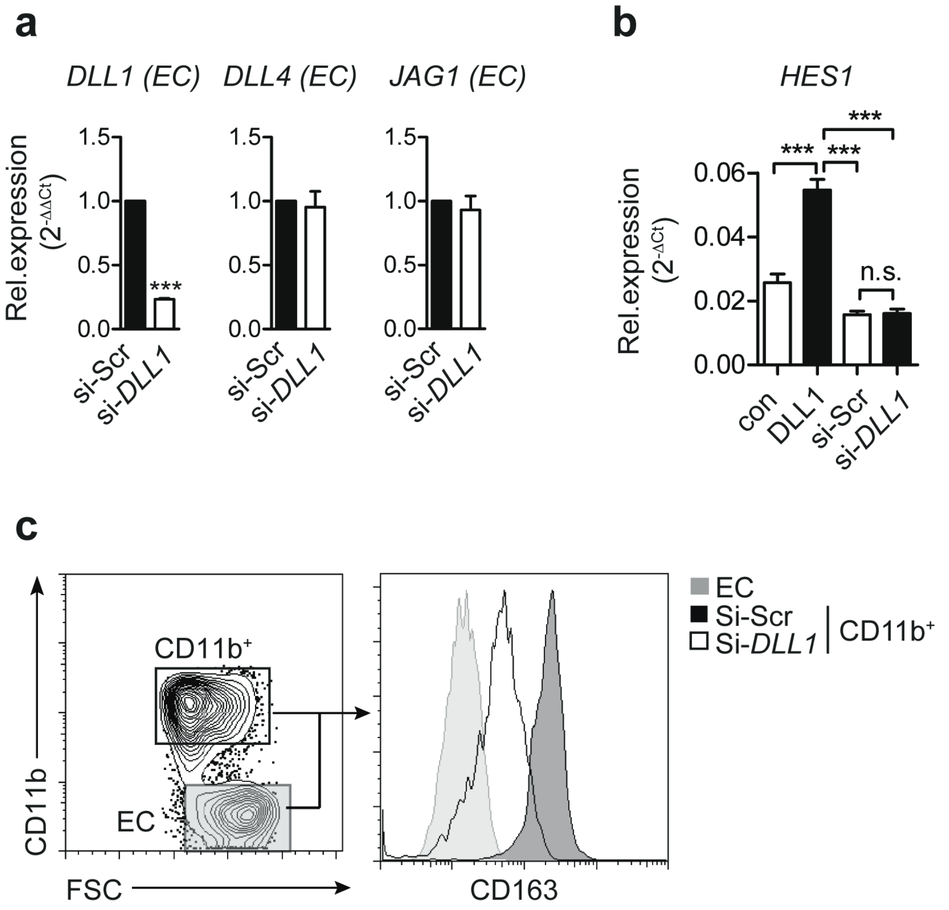

Supplementary Figure 11: Transfection of Arterial endothelial cells with si-DLL1 and analysis after co-culture with CD14$^+$ monocytes

(a) Quantitative RT-PCR analysis of expression of *DLL1, DLL4 and JAG1* in HAEC's after treatment with *DLL1* specific si-RNA (si-DLL1) or scrambled si-RNA (si-Scr). Data is cumulative of n=3 independent experiments, performed in duplicates, error bars represent s.e.m. ***p<0.001, student's paired t-test. **(b)** Quantitative RT-PCR analysis comparing expression of *HES1* and *NRARP* on Arterial endothelial cells cultured on DLL1 ligand to transfected endothelial cells (si-Scr and si-*DLL1*). Endothelial cells were cultured on IgG as control. n=3 independent experiments, error bars represent s.e.m. ***p<0.001,**p<0.01,*p<0.05, one way ANOVA with Bonferroni multiple comparison test. **(c)** Representative flow cytometry from d3 co-culture of human CD14$^+$ monocytes with HAEC after treatment with *DLL1* specific si-RNA (si-DLL1) or scrambled si-RNA (si-Scr). n= 2 independent experiments.

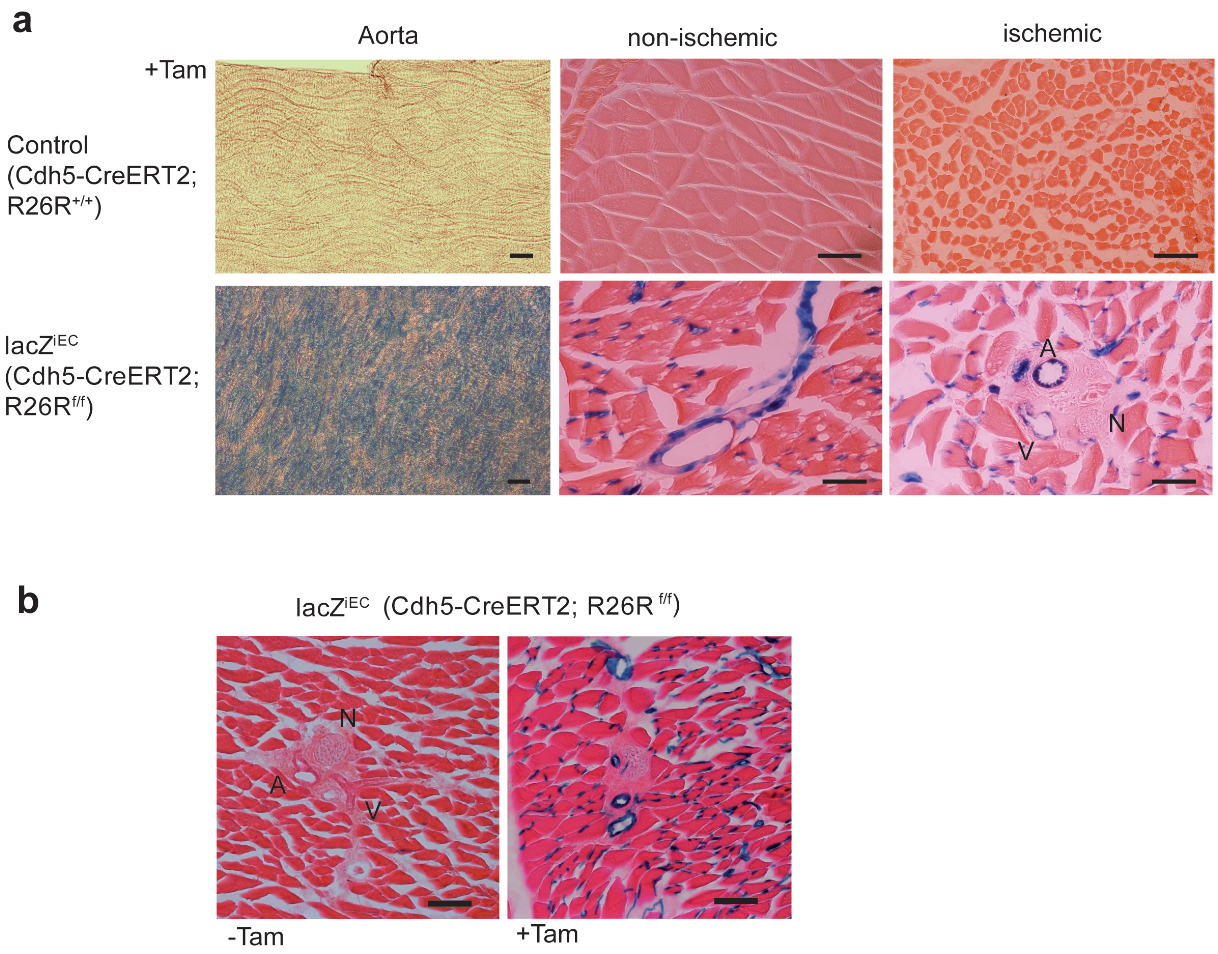

Supplementary Figure 12: Inducible *in vivo* EC targeting in lacZ^{iEC} Cre-reporter mice.

(a) β-galactosidase staining in lacZ^{iEC} and control mice after pulse of Tam in aorta (x10), baseline (non-ischemic) and ischemic muscle (x20). **(b)** Inducible β-galactosidase staining with and without Tam induction. Scale bar: 100μm.

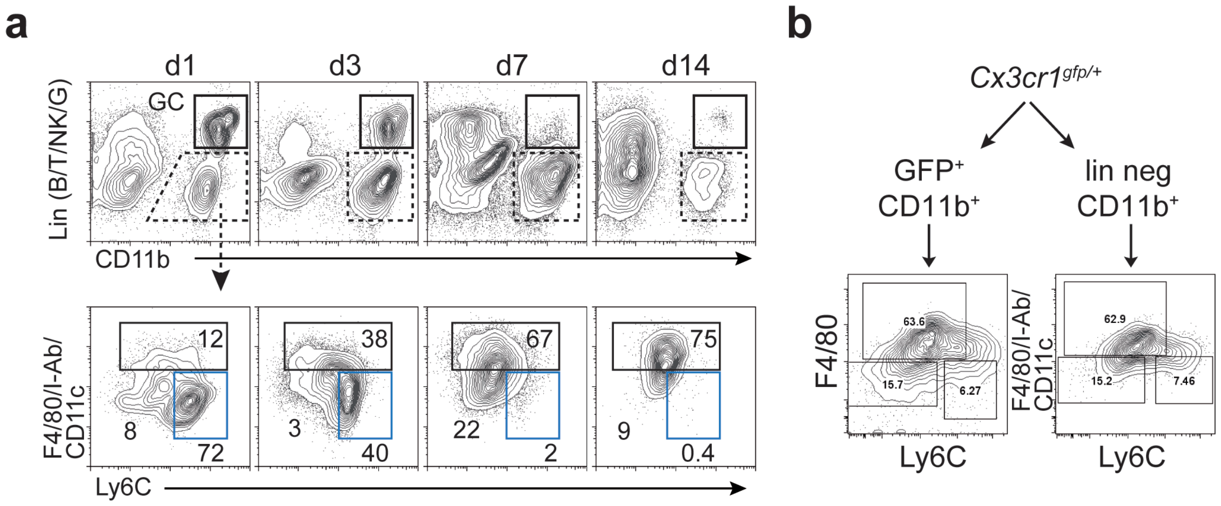

Supplementary Figure 13: Non-GFP based gating strategy for myleoid subset analysis

(a) Gating and timecourse of cell populations in ischemic muscle. The Lineage cocktail (lin) used for analysis includes a combination of the markers, B220/CD19/CD3ε/CD90/NK1.1/Ly6G (B/T/NK/G). After excluding granulocytes, ($lin^{+}CD11b^{+}$), the $lin^{neg}CD11b^{+}$ population is gated for $Ly6C^{hi}$ monocytes (blue box, $Ly6C^{hi}F4/80/I\text{-}A^{b}/CD11c^{low/neg}$ and macrophages (black box, $F4/80/I\text{-}A^{b}/CD11c^{+}$). **(b)** Representative comparison of two gating strategies for macrophages in the same *Cx3cr1*$^{gfp/+}$ mouse showing identical population frequencies for macrophages and $Ly6C^{hi}$ monocytes. n=3 independent experiments.

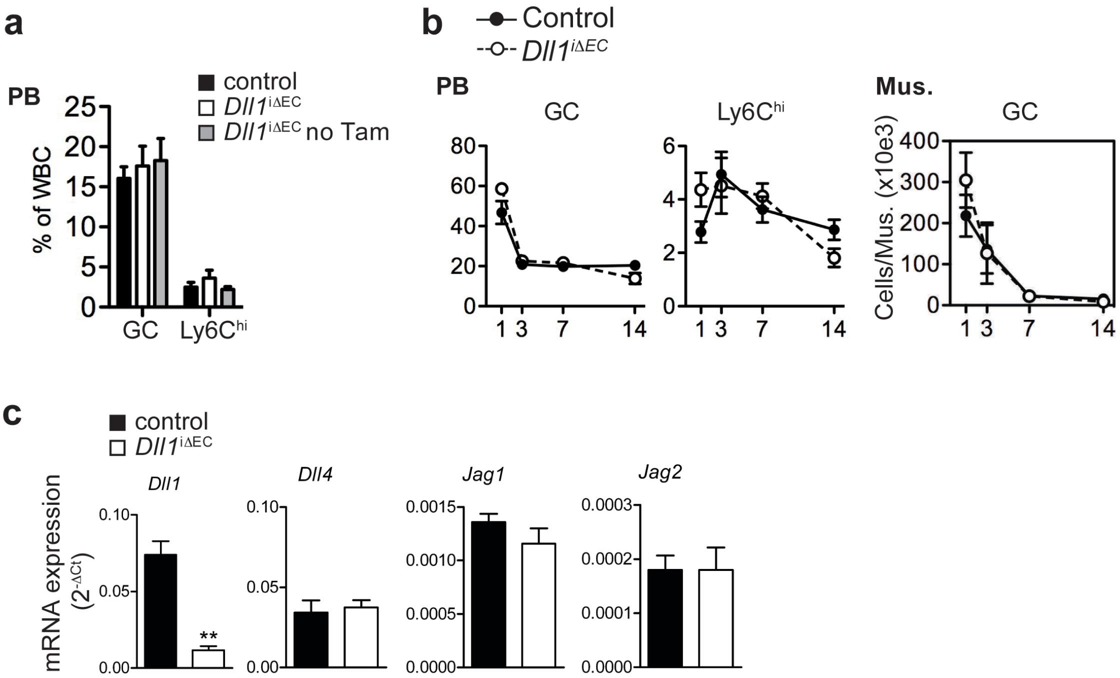

Supplementary Figure 14: Comparison of cell distribution kinetics and Notch ligand distribution between control and Dll1$^{i\Delta EC}$ mice after HLI

(a) Distribution of leukocytes depicted as percent of WBC in littermate control, *Dll1$^{i\Delta EC}$* and *Dll1$^{i\Delta EC}$* without tamoxifen. n=5/10/10 mice/group, error bars represent s.e.m. **(b)** Analysis of cell populations by flow cytometry in peripheral blood (PB) and ischemic muscle (Mus) of induced endothelial Dll1 mutant (*Dll1$^{i\Delta EC}$*) mice and control mice. n= 8 mice/group, error bars represent s.e.m. **(c)** Quantitative RT-PCR analysis of expression of Notch ligands from d3 ischemic muscle. Data is cumulative of n=3 mice/group, measurements performed in duplicates, error bars represent s.e.m. **p<0.01, student's unpaired t-test.

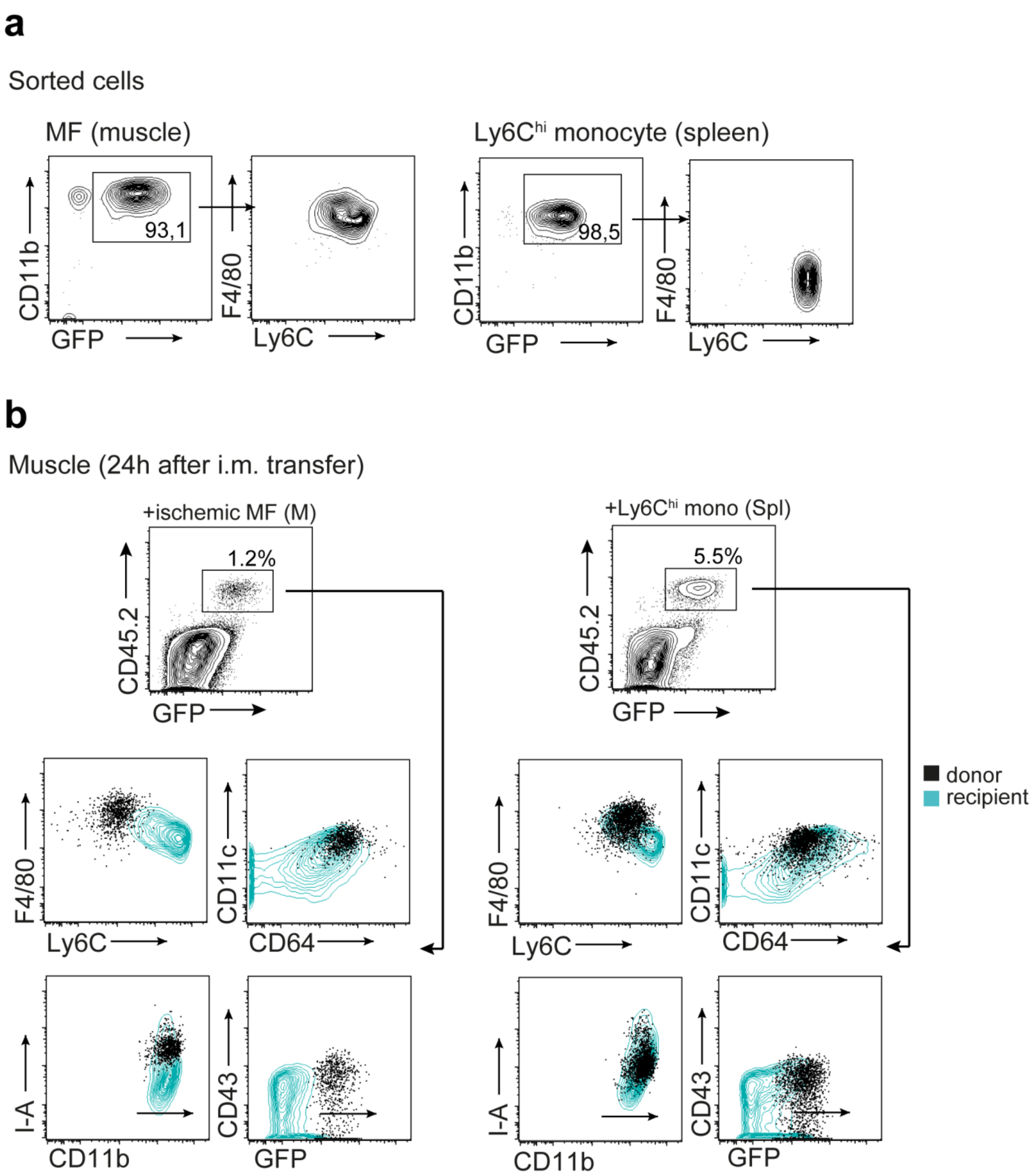

Supplementary Figure 15: Sorting strategy, analysis and recovery of donor cells after injection. Splenic monocytes or muscle macrophages (MF) were injected at a dose of $5x10^5$ cells into muscle at day 1 after HLI. Flow cytometry analysis was performed 24h after injection. **(a)** Expression of surface markers on sorted donor cells before adoptive transfer. **(b)** Recovery and cell fate tracking of transferred donor cells in the ischemic muscle post adoptive transfer.

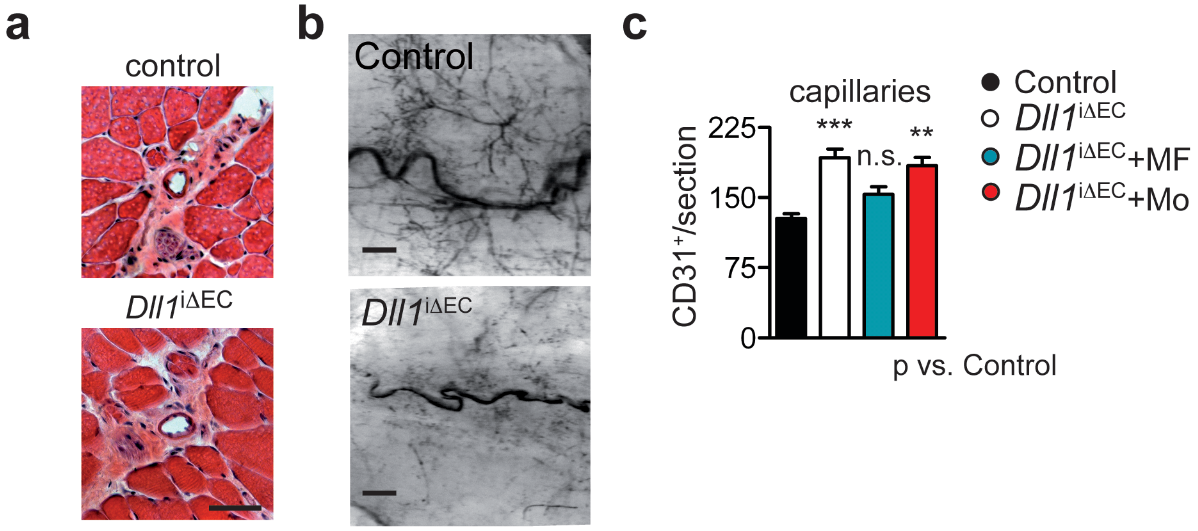

Supplementary Figure 16: Comparison of vessel architecture between control and Dll1^{iDEC} mice after HLI. **(a)** Representative H&E stained muscle sections of baseline collateral arteries. Scale bar 100 um. **(b)** Representative visualization of hind limb arteries and collateral branches in the iTA muscle (M) by pigment particle perfusion. Scale bar 1mm. **(c)** Quantification of CD31^{+} vessels per section d14, n= 5 mice/group, error bars represent s.e.m. *** p<0.001 vs control by one way ANOVA and Dunnett's multiple comparison test

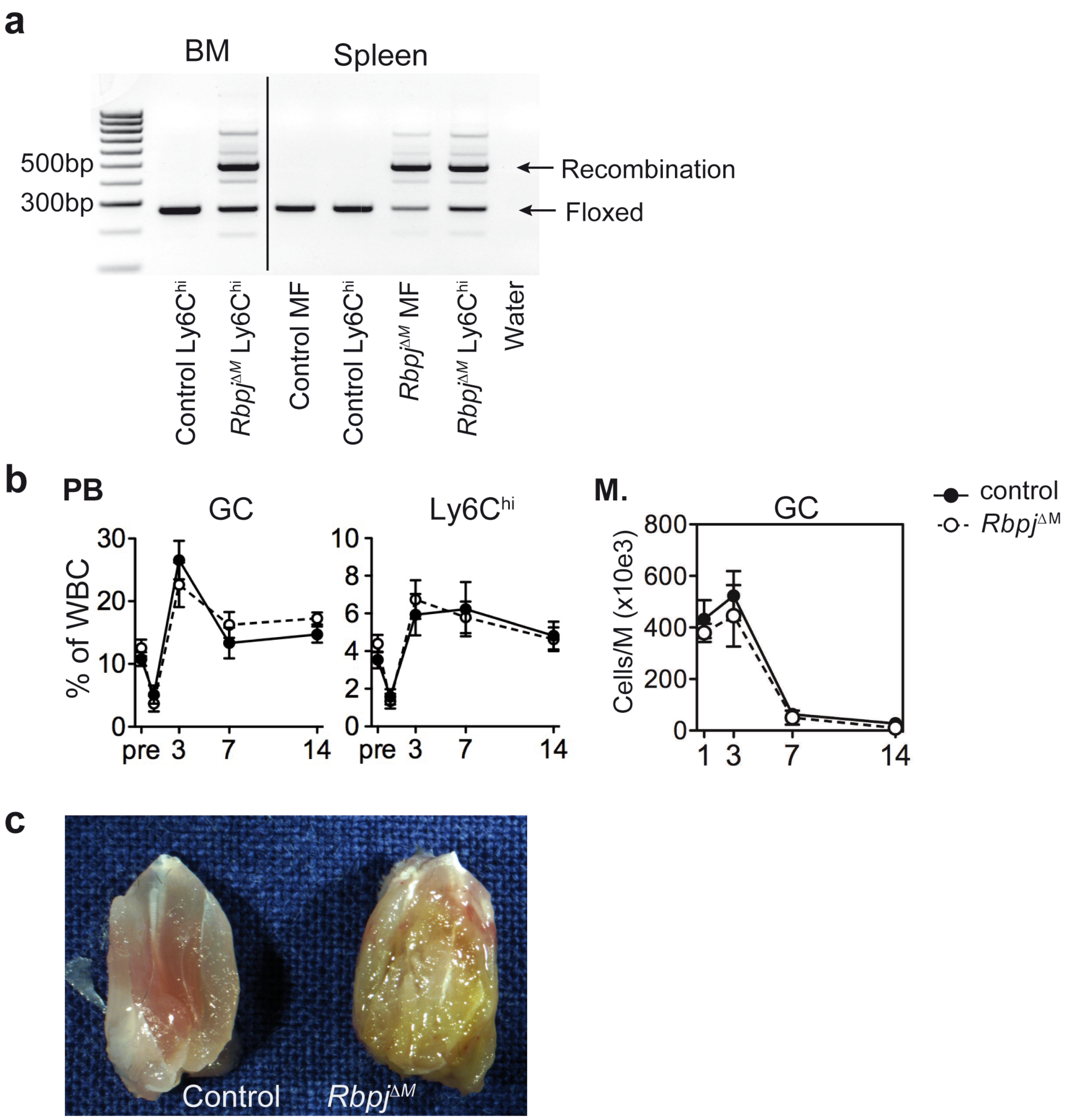

Supplementary Figure 17: Characterization of $Rbpj^{\Delta M}$ mice

(a) Agarose gel-electrophoresis of PCR products from isolated Ly6C^{hi} monocytes or macrophages (MF) showing Cre-mediated recombination in monocytes and macrophages. **(b)** Kinetics of Granulocytes, Ly6C^{hi} monocytes in the peripheral blood (PB) and muscle (M) of $Rbpj^{\Delta M}$ mice and littermate controls. n= 7/9 mice/group, error bars represent s.e.m. **(c)** Representative micrograph of d14 isolated gastrocnemius muscle from ischemic limb. Magnification: 5x.

Supplementary Table 1: Phenotypic characterization by FACS

Cell type	Phenotype	
	**Non-*Cx3cr1*-GFP strategy**	***Cx3cr1*-GFP strategy**
Granulocytes	(B220/CD19/CD3ε/CD90/NK1.1/ Ly6G)$^{+}$CD11b^{+}	(B220/CD19/CD3ε/CD90/NK1.1/ Ly6G)$^{+}$CD11b^{+}GFP^{neg}
Ly6C^{hi} monocytes	(B220/CD19/CD3ε/CD90/NK1.1/ Ly6G)^{neg}CD11b^{+}Ly6C^{hi} F4/80/I-A^{b}/CD11c$^{lo/neg}$	(B220/CD19/CD3ε/CD90/NK1.1/ Ly6G)^{neg}CD11b^{+}Ly6C^{hi} GFP^{+}F4/80$^{lo/neg}$
Ly6C^{lo} monocytes	(B220/CD19/CD3ε/CD90/NK1.1/ Ly6G)^{neg}CD11b^{+} Ly6C$^{lo/neg}$F4/80/I-A^{b}/CD11c$^{lo/neg}$	(B220/CD19/CD3ε/CD90/NK1.1/ Ly6G)^{neg}CD11b^{+} Ly6C$^{lo/-}$GFP^{hi}F4/80$^{lo/neg}$I-A^{b} ^{neg}CD11C^{+}CD43^{+}
Macrophages	(B220/CD19/CD3ε/CD90/NK1.1/ Ly6G)^{neg}CD11b^{+} F4/80/I-A^{b}/CD11c^{+}	(B220/CD19/CD3ε/CD90/NK1.1/ Ly6G)^{neg}CD11b^{+} GFP^{+}F4/80^{hi}
Dendritic cells (DC)		(B220/CD19/CD3ε/CD90/NK1.1/ Ly6G)^{neg}CD11b^{+} GFP^{lo}F4/80^{lo}CD11c^{+}I-A$^{b\ hi}$

Lineage markers, Lin are B220/CD19/CD3ε/CD90/NK1.1/Ly6G

Supplementary Table 2: Genetic strains used in the study

Name	Mouse description	Mouse background
GFP^{+}	*Cx3cr1*$^{GFP/+}$	*B6*
Control	*LysM*$^{+/+}$*Rbpj*$^{lox/lox}$*Cx3cr1*$^{GFP/+}$	*B6*
Rbpj$^{\Delta M}$	LysM$^{Cre/+}$Rbpj$^{lox/lox}$Cx3cr1$^{GFP/+}$	*B6*
CD45.1^{+}	B6.SJL-*Ptprc*^{a}*Pepc*^{b}/BoyJ	*B6*
Control	*Gt(ROSA)26Sor*	*B6*
lacZ^{iEC}	*Cdh5(BAC)-CreERT2 Gt(ROSA)26Sor*	*B6*
Control	*Dll1*$^{lox/lox}$	*Mixed, B6;129*
Dll1$^{i\Delta EC}$	*Cdh5(BAC)-CreERT2 Dll1*$^{lox/lox}$	*Mixed, B6;129*
Dll1$^{+/LacZ}$	*Dll1*$^{+/LacZ}$	*129*

Supplementary Table 3: Murine RT-PCR primers used in the study

Gene	Primer pair
Rps9	Forward: GGA TTT CTT GGA GAG GCG GC<br>Reverse: ACC TGC TTG CGG ACC CTA AT
Etv3	Forward: TCG CTC GAG TGG TGT GGT TC<br>Reverse: GTG AGC TGG CAG AGA AGCG A
MafB	Forward: CGC GAG AGA GAC GCC TAC AA<br>Reverse: CCC GCC AGG ACT CAC AGA AA
Mrc1	Forward: CTC GTG GAT CTC CGT CAC AC<br>Reverse: GCA AAT GGA GCC GTC TGT GC
Myc	Forward: GCT GTT TGA AGG CTG GAT TTC<br>Reverse: GAG TCG TAG TCG AGG TCA TAG T
Sra	Forward: GCA AAG CAA CAG GAG GAC ATC A<br>Reverse: CTG TTC CAC GTG CGC TTG TT
Cxcl1	Forward: TAA CCA GTT CCA GCA CTC CAG ACT<br>Reverse: TGT TCT TGA GGT GAA TCC CAG CCA
Cxcl2	Forward: ACA TCC CAC CCA CAC AGT GAA AGA<br>Reverse: TCC TTC CAT GAA AGC CAT CCG ACT
Il12	Forward: ACC TGC TGA AGA CCA CAG ATG ACA<br>Reverse: TAG CCA GGC AAC TCT CGT TCT TGT
Il10	Forward: GGG TTG CCA AGC CTT ATC GGA AAT<br>Reverse: TCT TCA GCT TCT CAC CCA GGG AAT
Il6	Forward: ATC CAG TTG CCT TCT TGG GAC TGA<br>Reverse: TAA GCC TCC GAC TTG TGA AGT GGT
Hes1	Forward: CCG GAC AAA CCA AAG ACG GC<br>Reverse: GGA ATG CCG GGA GCT ATC TTT CT
Hey1	Forward: GCG CGG ACG AGA ATG GAA AC<br>Reverse: GGC GCT TCT CGA TGA TGC CT
Hey2	Forward: TGA AGC GCC CTT GTG AGG AA<br>Reverse: TTG TAG CGT GCC CAG GGT AA
Dll1	Forward: TCC GAT TCC CCT TCG GCT TC<br>Reverse: TGG GTT TTC TGT TGC GAG GT
Ets2	Forward: TGG CAT CCC CAA AAA CCC CT<br>Reverse: GCC GTT CAT GCC AAA CTG GT
Mertk	Forward: CGC TCT GGA GTG GAG GCA C<br>Reverse: GCC TGT GGT TGA CTG GGA GT
Spi1	Forward: TGA TGG AGA AGC TGA TGG CTT GGA<br>Reverse: TGC TTG GAC GAG AAC TGG AAG GTA
Cdk1	Forward: CCC GGC GAG TTC TTC ACA GA<br>Reverse: CCA CAG CGT CAC TAC CTC GT
Cdk2	Forward: TTG GAG AGG GCA CGT ACG GA<br>Reverse: TTC AGT CTC AGT GTC GAG CCG

Arg1	Forward: GTC CCT AAT GAC AGC TCC TTT C<br>Reverse: CCA CAC TGA CTC TTC CAT TCT T
Nos2	Forward: TGG TGG TGA CAA GCA CAT TTG GGA<br>Reverse: TGT CAT GAG CAA AGG CGC AGA ACT
Zbtb46	Forward: AGA GAG CAC ATG AAG CGA CA<br>Reverse: CTG GCT GCA GAC ATG AAC AC
Dll4	Forward: GGC CGG GAA CCT TCT CAC TC<br>Reverse: TTT CCT GGC GAA GTC TCT GGC
Jag1	Forward: CAA ATG AGT GCG AGG CCA AAC CTT<br>Reverse: AGC CAG GAA GGC AAT CAC AGT AGT
Jag2	Forward: CAA TGA CAC CAC TCC AGA TGA G<br>Reverse: GGC CAA AGA AGT CGT TGC G

Supplementary Table 4: Human RT-PCR primers used in the study

Gene	Primer pair
RPS9	Forward: TGG TTT GCT TAG GCG CAG AC<br>Reverse: CCG CGG GGT CAC ATA AGT TT
ETV3	Forward: CAG AAG GAG GTG GAG GGT ATC A<br>Reverse: CGA TGA CAT GGC GGA ACT CT
MAFB	Forward: CAA CGA CTT CGA CCT GCT CAA<br>Reverse: CGG AGC TAC ACG GAG TGC TG
MYC	Forward: GTA GTG GAA AAC CAG CAG CCT C<br>Reverse: GTT CTC CTC CTC GTC GCA GTA
HES1	Forward: CAC GAC ACC GGA TAA ACC AAA G<br>Reverse: CGC GAG CTA TCT TTC TTC AGA G
NRARP	Forward: ACA CTG CGT GGT CAA TGT GG<br>Reverse: CAG GCT GGG CGG TAT TTT CA
IL10	Forward: TCC TTG CTG GAG GAC TTT AAG GGT<br>Reverse: TGT CTG GGT CTT GGT TCT CAG CTT
IL8	Forward: AGC CTT CCT GAT TTC TGC AGC TCT<br>Reverse: AAT TTC TGT GTT GGC GCA GTG TGG
CSF1	Forward: TAC TGT AGC CAC ATG ATT GGG A<br>Reverse: CCT GTG TCA GTC AAA GGA AC
CSF2	Forward: CAC TGC TGC TGA GAT GAA TGA A<br>Reverse: GTC TGT AGG CAG GTC GGC TC
DLL1	Forward: GAG CGT GGG GAG AAA GTG TG<br>Reverse: TCT GCA CTT GCA TTC CCC TG
DLL4	Forward: ATC AGC GAT ATG CTC CCC CA<br>Reverse: TGC CTT ATA CCT CCG TGG CA
JAG1	Forward: GTA GCA ACA CAG GCC CTG AC<br>Reverse: AGG CGT GCT CAG CAA TTT CA
HEY1	Forward: AGG CTG GTA CCC AGT GCT TT<br>Reverse: GCG CGT CAA AGT AAC CTT TCC

Supplementary Table 5: Antibodies and fluorescence dyes for flow cytometry and immunostaining

Antibody	Clone	Dilution	Company
Anti-mouse CD3ε	145-2C11	1:100	BioLegend
Anti-mouse CD45R/B220	RA3-6B2	1:400	BioLegend
Anti-mouse Ly6G	1A8	1:400	eBioscience
Anti-mouse CD19	1D3	1:400	BD Pharmingen
Anti-mouse CD90	53-2.1	1:500	BD Pharmingen
Anti-mouse NK1.1	PK136	1:400	BioLegend
Anti-mouse CD115	AFS98	1:100	Biolegend
Anti-mouse/human CD11b	M1/70	1:400	BioLegend
Anti-mouse Ly6C	HK1.4	1:2800	BioLegend
Anti-mouse F4/80	BM8	1:100	BioLegend
Anti-mouse CD11c	N418	1:400	BioLegend
Anti-mouse CD45.2	104	1:200	BioLegend
Anti-mouse CD64	X54-5/7.1	1:400	BioLegend
Anti-mouse I-Ab	AF6-120.1	1:100	eBioscience
Anti-mouse CD45	30-F11	1:100	BD Pharmingen
Anti-mouse CD31	MEC 13.3	1:100	BD Pharmingen
Rabbit anti-GFP	SP3005P	1:200	Acris GmbH
Anti-mouse CD284 (TLR4)	SA 15-21	1:100	Biolegend
Anti-mouse CD43	S7	1:400	BD Pharmingen
Anti-human CD14	MφP9	1:400	BD Pharmingen
Anti-human CD16	B73.1	1:100	BD Pharmingen
Anti-human CD163	GHI/61	1:100	BD Pharmingen
Anti-human CD206	19.2	1:100	BD Pharmingen
Anti-human CD11b	ICRF44	1:400	BD Pharmingen
Anti-human CD209	DCN46	1:100	BD Pharmingen
Anti-human CD86	2331	1:100	BD Pharmingen
Anti-human HLA-DR	L243	1:400	BioLegend
Streptavidin PerCP		1:100	BD Pharmingen
Anti-mouse SMA-Cy3™	1A4	1:100	Sigma Aldrich
Alkaline phosphatase Anti-Rat IgG (H+L)	Polyclonal	1:200	AbCam
FITC Affinipure Goat Anti-Rabbit IgG (H+L)	Polyclonal	1:200	Jackson ImmunoResearch
Cy3™ Affinipure Donkey Anti-Rat IgG (H+L)	Polyclonal	1:100	Jackson ImmunoResearch
AnnexinV		1:50	BioLegend
7AAD		1:100	BioLegend

DAPI		1:5000	Invitrogen
Propidium Iodide		1:12000	Sigma
